# Epithelial membrane protein 1 drives hepatic stellate cell activation via the TLN1/FAK cascade in MASLD donor liver transplantation

**DOI:** 10.1186/s43556-025-00371-7

**Published:** 2025-11-24

**Authors:** Tongxi Li, Ran Liu, Huan Cao, Shenghe Deng, Gengqiao Wang, Xueling Wang, Peng Zhao, Xuan Li, Jingjin Zhu, Shuyu Shao, Hao Chen, Lei Liu, Chen Zhang, Chuanzheng Yin, Zifang Song

**Affiliations:** 1 Department of Hepatobiliary Surgery, Union Hospital, Tongji Medical College, Huazhong University of Science and Technology, Wuhan, Hubei China; 2 Center for Liver Transplantation, Union Hospital, Tongji Medical College, Huazhong University of Science and Technology, Wuhan, Hubei China

**Keywords:** Metabolic dysfunction-associated steatotic liver disease, Ischemia/reperfusion injury, Liver Transplantation, Epithelial membrane protein 1, Hepatic Stellate Cell

## Abstract

The prevalence of metabolic dysfunction-associated steatotic liver disease (MASLD) has significantly increased, prompting the increased use of steatotic donor livers in transplantation, contributing to a higher incidence and severity of ischemia–reperfusion injury (IRI), necessitating the development of targeted interventions for MASLD-related liver transplantation (MASLD-IRI). Here, we identified epithelial membrane protein 1 (EMP1) as a potential diagnostic and therapeutic target in MASLD-IRI using multi-omics analysis and mechanistic investigations in rodent models and cells, further validating our findings in human samples. Phenotypic observations revealed significant activation of hepatic stellate cells (HSCs) under MASLD-IRI conditions, leading to increased inflammatory liver injury, which correlated with significant upregulation of EMP1 in HSC. Mechanistically, EMP1 upregulation inhibited SMAD-specific E3 ubiquitin-protein ligase 1 (SMURF1)-mediated ubiquitination and degradation of talin1 (TLN1) by competing with SMURF1 for the TLN1 binding site. The subsequent accumulation of TLN1 further promoted phosphorylation of focal adhesion kinase (FAK), establishing a pro-inflammatory signaling axis—EMP1/TLN1/FAK—that amplified HSC activation and aggravated liver injury. Silencing EMP1 suppressed the TLN1/FAK post-translational modification cascade, thereby attenuating HSC activation and downstream inflammation. These findings highlight the potential of EMP1 as a biomarker to monitor the prognosis of MASLD transplantation, as well as a therapeutic target to improve prognosis.

**Supplementary Information:**

The online version contains supplementary material available at 10.1186/s43556-025-00371-7.

## Introduction

Liver transplantation (LT) remains the definitive treatment for end-stage liver disease; however, its clinical impact is limited by the shortage of donor organs. Marginal grafts are increasingly incorporated in current strategies to expand donor pools, and steatotic livers now constitute > 30% of the transplanted organs in some centers [1]. This paradigm shift is attributed to the increased prevalence of metabolic dysfunction-associated steatotic liver disease (MASLD) that contributes to the increased use of steatotic donor livers in transplantation [2]. However, clinical evidence suggests that even mild steatosis leads to early graft dysfunction (EAD) due to increased susceptibility to ischemia/reperfusion injury (IRI) [3–5]. These pathophysiological interactions highlight the need for mechanistic investigations into MASLD-IRI pathogenesis, particularly given the expanding utilization of steatotic grafts in clinical practice.

Hepatic stellate cells (HSCs) are central drivers of hepatic fibroinflammatory injury, and their activation is tightly coupled with liver inflammation through a self-amplifying loop. Although quiescent HSCs maintain homeostasis, pathological conditions such as MASLD and IRI trigger their activation through shared pathways [6–8]. This process is exacerbated by a pro-inflammatory microenvironment consisting of injured cells that release cytokines and damage-associated molecular patterns (DAMPs) that stimulate HSCs, which in turn further secrete inflammatory mediators and extracellular matrix proteins, thereby perpetuating liver injury [9, 10]. Single-cell studies further link early HSC activation signatures to IRI severity [11]. Moreover, focal adhesion protein kinase (FAK) plays a critical role as a key integrator of mechanosignaling and soluble signaling in reprocessing. FAK activation enhances HSC proliferation, migration, and inflammatory output, thereby accelerating fibrosis and inflammation [12]. Thus, targeting FAK may disrupt this self-amplifying loop and alleviate disease progression.

Epithelial membrane protein 1 (EMP1)—a member of the tetraspanin family known for its roles in tumor-stroma interactions [13]—possesses four conserved transmembrane domains and cysteine-rich extracellular loops that facilitate dynamic protein interactions, thereby modulating cell adhesion and signaling processes [14]. Under physiological conditions, EMP1 is predominantly expressed in the gastrointestinal tract, skin, lungs, brain, and bile ductules, with relatively low expression in the liver. However, emerging evidence indicates that EMP1 expression is upregulated in various forms of liver injury and fibrotic diseases, suggesting its potential role as a biomarker for liver fibrosis. Notably, EMP1 expression is significantly elevated in activated HSCs compared to that in quiescent HSCs [15]. This upregulation may be functionally associated with the previously reported roles of EMP1 in promoting cancer cell proliferation, migration, and metastasis, as well as its involvement in fibroblast activation and extracellular matrix remodeling—key processes in HSC biology and fibrogenesis [15, 16]. Given these properties, EMP1 could play significant roles in liver pathology, particularly in the context of metabolic and ischemic stress responses. However, the precise contributions of EMP1 to liver pathophysiology require further investigation. While studies have shown that EMP1 expression is induced by pro-fibrotic mediators such as TGF-β, PI3K/AKT, and NF-κB, and in response to mechanical stress, the detailed mechanisms underlying EMP1 expression regulation in HSCs remain incompletely understood [17–20].

In this study, we used animal and cell models to evaluate the role of EMP1 in HSC activation during MASLD-IRI-associated injury. We demonstrate that EMP1 coordinates the post-translational regulation of the talin-1 (TLN1)/FAK axis by competitively interfering with ubiquitination, establishing a novel signaling paradigm in liver injury. Our study highlights the clinical significance of EMP1 in MASLD donor liver transplantation as both a prognostic biomarker and a therapeutic target, offering actionable strategies to improve postoperative management of steatotic grafts.

## Results

### MASLD donor livers exacerbate graft IRI through HSC activation

To investigate the impact of donor liver type on IRI in liver transplantation, we established an MASLD donor model by using a high-fat diet (HFD) to mimic donor MASLD conditions and by inducing IRI via LT (ND/HFD-Sham/LT composite model) (Fig. S1a). The successful induction of the MASLD donor model was confirmed through lipid profiling (Fig. S1b–d).

**Fig.1:**
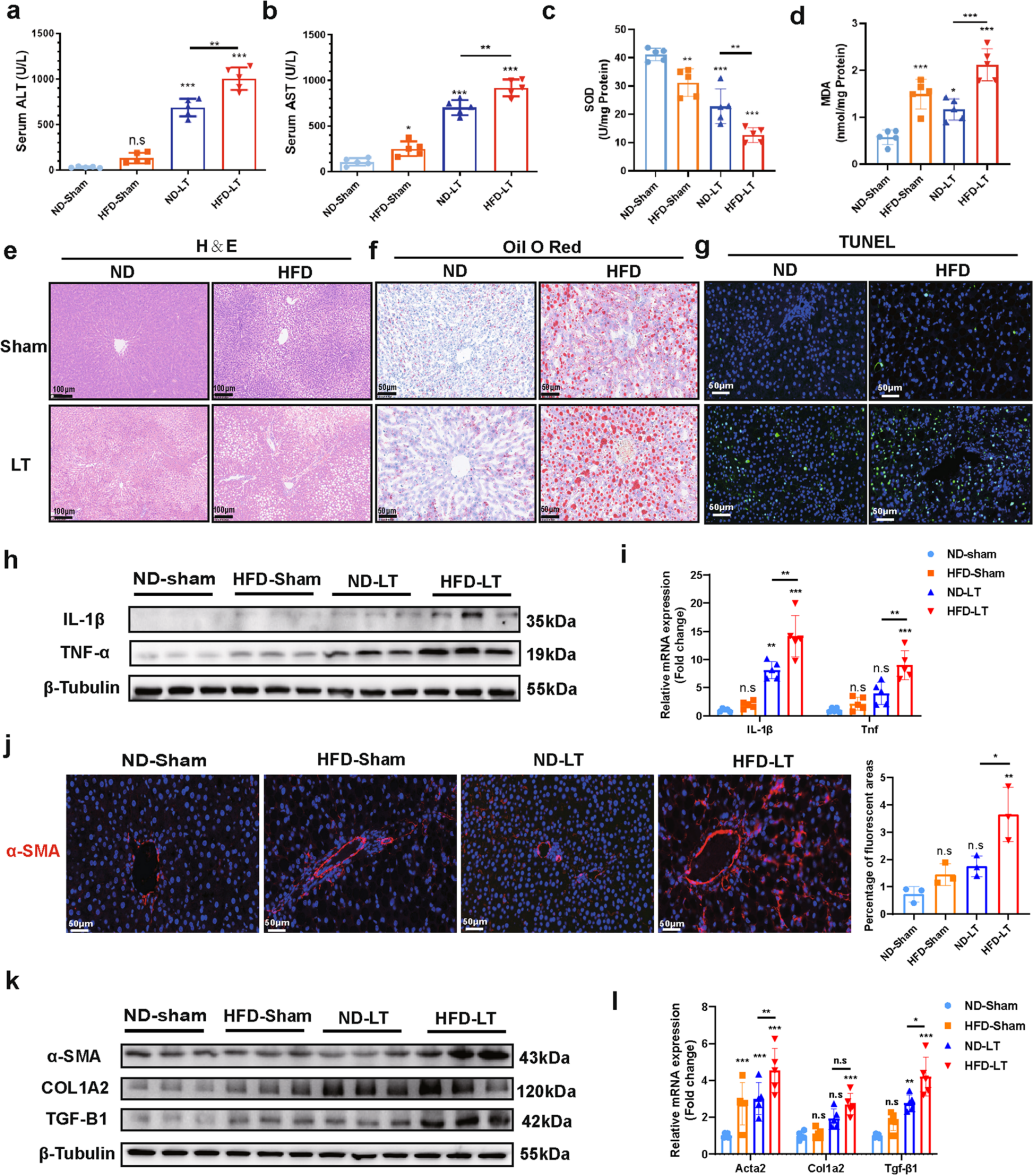
MASLD donor livers exhibit more severe IRI than normal donor livers in a rat transplantation model. ND-Sham group: untreated control group; HFD-Sham group: group fed a high-fat diet constructed MASLD donor and treated with sham surgery to obtain samples directly from the donor; ND-LT: donor on a normal diet and underwent an OLT procedure, and finally samples were obtained in the recipients; HFD-LT: donor fed a high-fat diet constructed MASLD donor and underwent an OLT surgery and finally samples were obtained in the recipient. **a**, **b**. Serum levels of liver injury markers, alanine aminotransferase (ALT) and aspartate aminotransferase (AST), were measured to evaluate the overall extent of hepatic damage (*n* = 5/group). **c**, **d**. Hepatic levels of oxidative stress markers, superoxide dismutase (SOD) and malondialdehyde (MDA), were detected to assess oxidative injury (*n* = 5/group). **e–g**. Histopathological examination of rat liver tissues was performed to characterize the injury phenotype. H&E staining (Scale bar = 100 μm) revealed hepatic edema, structural disarray, and necrotic areas. Oil red O staining (Scale bar = 50 μm) shows lipid accumulation. TUNEL staining(Scale bar = 50 μm) was used to evaluate hepatocyte apoptosis across different models. for all pathology stained images *n* = 3/group. **h**, **i**. Protein(*n* = 3/group) and mRNA(n = 5/group) expression levels of classical inflammatory cytokines, IL-1β and TNF-α, were measured in liver tissues to reflect the severity of inflammation. **j**. Immunofluorescence staining of α-SMA, a marker of HSC activation, was conducted to evaluate the activation status of stellate cells. Scale bar = 50 μm (*n* = 3/group). **k**, **l**. Protein(*n* = 3/group) and mRNA(*n* = 5/group) expression of multiple Hepatic stellate cell activation markers were detected.The data used for the above statistics were SD ± mean, n.s > 0.05, * *P* < 0.05, ** *P* < 0.01, *** *P* < 0.001, and analyzed by two-way ANOVA or t-test followed by Tukey's test

Serum analysis revealed significantly elevated levels of alanine aminotransferase (ALT) and aspartate aminotransferase (AST) in the HFD-LT group compared with those in ND-sham, HFD-Sham, and ND-LT groups (Fig. 1a,b). The oxidative stress markers superoxide dismutase (SOD) and malondialdehyde (MDA) similarly showed enhanced oxidative damage in the HFD-LT group compared to that in the ND-LT group (Fig. 1c,d). Pathological assessments including hematoxylin and eosin (H&E), oil red O, and TUNEL staining demonstrated more severe structural disruption, increased lipid accumulation, and elevated cell death in the HFD-LT group (Fig. 1e–g), indicating that MASLD donor livers aggravate IRI during transplantation. Further evaluation of classical inflammatory cytokines including interleukin (IL)−1β and tumor necrosis factor-α (TNF-α) revealed a stronger inflammatory response in the HFD-LT group (Fig. 1h,i), implicating the involvement of inflammatory pathways in exacerbating liver injury.

Prior studies have indicated that combined HFD and IRI can induce sterile inflammation and liver injury, where HSCs play a critical role [9, 20–22]. We therefore investigated HSC activation mechanisms. Expression of α-SMA, a perisinusoidal HSC activation marker, was significantly upregulated in the HFD-LT group (Fig. 1j). Protein and mRNA analyses further confirmed upregulation of multiple HSC activation markers (Fig. 1k,l), indicating that HFD-LT-modeled MASLD-IRI induced broad HSC activation, which may cause aseptic inflammation and fibrosis. This finding explains the observed elevation of inflammatory factors; however, subsequent analyses revealed that HFD-LT did not rapidly induce hepatic fibrosis (Fig. S2a,b). Moreover, the expression of ECM1—an inhibitor of extracellular matrix (ECM) synthesis was decreased [23], while that of CD44—involved in synthesis of the main ECM component hyaluronic acid—was elevated (Fig. S2c,d). ELISA further confirmed reduced ECM1 and increased hyaluronic acid levels (Fig. S2e,f), indicating altered ECM regulation. This suggests that MASLD-IRI may mediate longer-term susceptibility to fibrosis.

In summary, using MASLD donor livers significantly exacerbated IRI, likely through HSC activation and subsequent inflammatory release, without acute fibrotic formation. These findings highlight the need to identify key regulatory targets in HSC activation under MASLD-IRI conditions.

### EMP1 as a potential diagnostic and therapeutic target for MASLD-IRI

**Fig.2:**
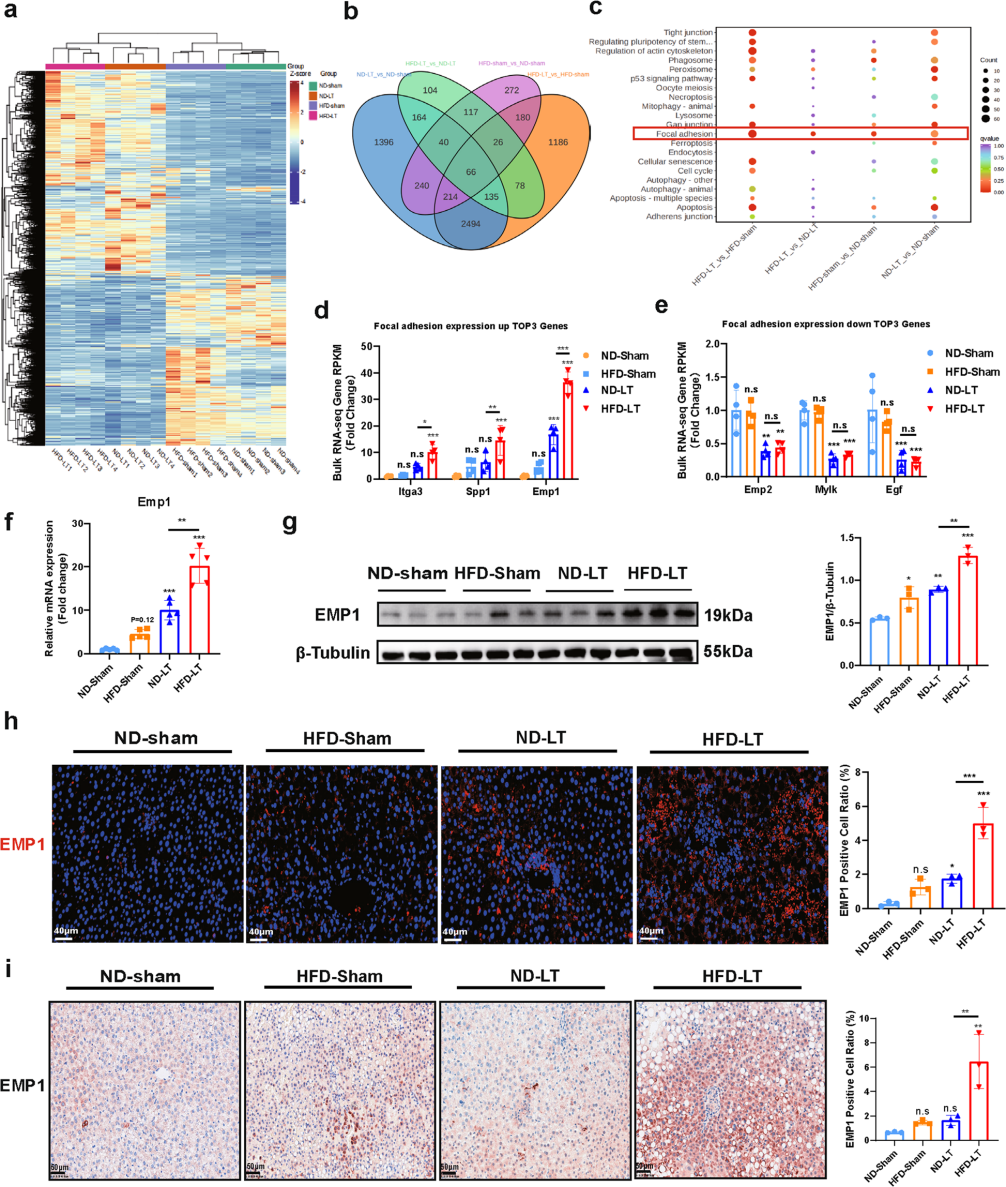
EMP1 expression was significantly upregulated in MASLD-IRI. The data in Fig. 2 follows the animal model constructed in Fig. 1.**a**, **b**. Transcriptome sequencing was performed on liver tissues of animals in each group and mRNA datasets were obtained for data analysis, heatmaps and Wayne plots showed the great differences in transcriptional levels between groups (*n* = 4/group). **c**. Differential gene enrichment analysis based on the KEGG database was performed to show the differential signaling pathways in the comparison between groups, and to excavate genes and signaling pathways that might be affected by both MASLD and IRI. **d**, **e**. Focal adhesion(FA) as the signaling pathway with the highest number of differential genes for comparisons between groups and comparisons between HFD-LT and ND-LT, we used the RPKM values of genes from transcriptome sequencing as the gene expression quantities and demonstrated changes in expression of the three genes with the most pronounced up-and down-regulation (*n* = 4/group). **f**, **g**. EMP1 as the most significant differential gene for comparison between groups in the Liver tissues were subjected to mRNA (n = 5/group), protein level (*n* = 3/group) expression validation. **h**, **i**. Immunofluorescence (Scale bar = 40 μm) and Immunohistochemistry staining (Scale bar = 50 μm) of liver tissue EMP1 was performed to observe its expression and expression localization(n = 3/group). All data used for statistics above are SD ± mean, n.s > 0.05, * *P* < 0.05, ** *P* < 0.01, *** *P* < 0.001, analyzed by two-way ANOVA or t-test followed by Tukey test

To identify potential therapeutic targets for MASLD-IRI, we conducted bulk RNA sequencing analysis (RNA-seq), which revealed distinct transcriptomic profiles across groups (Fig. 2a). Comparative analysis of differentially expressed genes (DEGs) among ND/HFD-Sham/LT groups was performed to identify consistent alterations associated with MASLD and IRI, with the goal of discovering dual-effective therapeutic targets (Fig. 2b; Fig. S3a–f). Kyoto Encyclopedia of Genes and Genomes (KEGG) enrichment analysis highlighted the focal adhesion (FA) pathway as significantly enriched based on the number of involved genes and magnitude of change (Fig. 2c), implicating its role in MASLD-IRI-induced liver injury. These results suggest that the FA pathway and its key kinase—focal adhesion kinase (FAK)—may represent a common therapeutic target for MASLD-IRI, consistent with prior studies linking FA signaling to HSC activation [24, 25].

Among the DEGs within the FA pathway, Emp1, Spp1, and Itga3 were significantly upregulated, while Egf, Mylk, and Emp2 were downregulated. Among these, only the three upregulated genes showed significant differences between HFD-LT and ND-LT groups, with Emp1 exhibiting the most pronounced change (Fig. 2d,e). We therefore focused on EMP1 and confirmed that its expression was significantly elevated in the unifactorial and multifactorial ND/HFD-Sham/LT groups in a gradient (Fig. 2f-i). Although EMP1 is an adhesion protein implicated in tumor invasion and migration via cell adhesion and transmembrane trafficking [12, 13, 15, 16], its role in non-oncological contexts such as liver disease remains poorly understood. Further investigation into EMP1 may elucidate the mechanisms underlying its upregulation in MASLD-IRI and clarify its biological function, thereby supporting its potential as a diagnostic and therapeutic target.

### EMP1 localizes in hepatic nonparenchymal cells and regulates HSC activation

**Fig.3:**
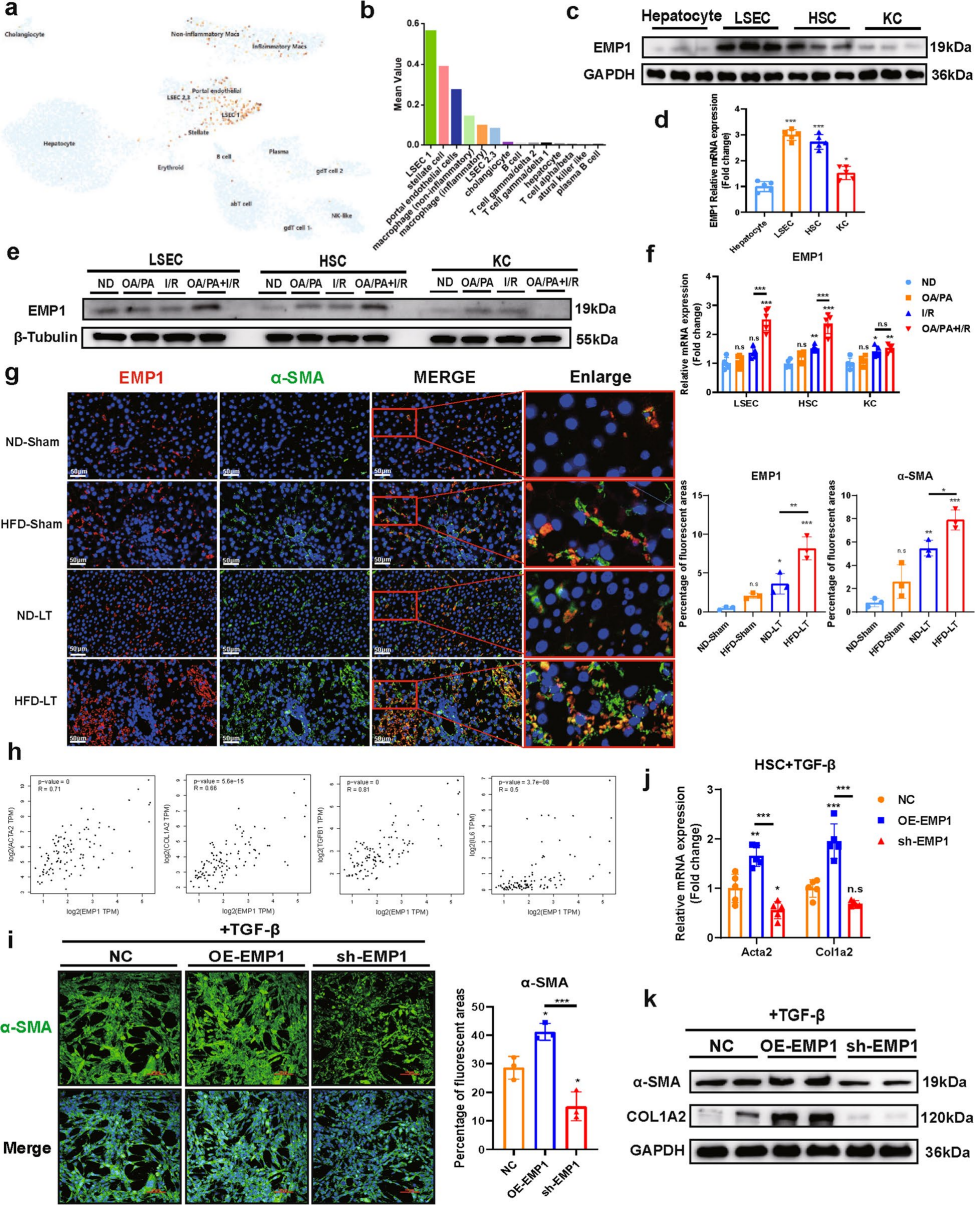
EMP1 localizes to hepatic non-substantive cells and induces HSC activation. ND: untreated control group; OA/PA: group using oleic acid (OA) + palmitic acid (PA) to simulate MASLD environment; I/R: group treated with hypoxia/reoxygenation incubator to simulate IRI; OA/PA + I/R: group simulating MASLD environment and treated with IRI. **a**, **b**. Cellular localization of EMP1 in the liver was resolved using the UCSC CellBrowser single-cell sequencing database, and it was found that EMP1 was mainly localized to hepatic non-substantive cells, especially endothelial cells, stellate cells, and inflammatory macrophages. **c**, **d**. Protein (*n* = 3/group) and mRNA (*n* = 5/group) expression of EMP1 in different LNPCs cell lines under OA/PA + IRI. **e**, **f**. Protein (*n* = 3/group) and mRNA (*n* = 5/group) expression of EMP1 in LNPCs cell lines under different intervention conditions of single (OA/PA or IRI) and combined (OA/PA + IRI) injury. **g**. By double immunofluorescence staining of hepatic tissues with EMP1, α-SMA, and α-SMA, it was observed that EMP1 co-localized with activated HSC and showed a co-regulation in expression (*n* = 3/group). SMA double immunofluorescence staining, it was observed that EMP1 was co-localized with activated HSC and co-expressed with up-regulation of expression (*n* = 3/group). Where red is EMP1, green is α-SMA, and blue is DAPI cell nuclei. The scale bar = 50 μM, shown in the lower left corner, and the red box shows the local zoomed image, where the yellow part is red + green co-localized EMP1 in activated HSC. **h**. Correlation of EMP1 with the expression of HSC activation markers and inflammatory factors in the liver as demonstrated by raw letter analysis of the GEPIA2 database. **i.** Immunofluorescence staining was performed to detect α-SMA, under the overexpression or silencing of EMP1. α-SMA is shown in green, and the nuclei of the cells in blue with DAPI, which was labeled as a red scale bar due to the high staining luminance. The scale bar = 100 μm (*n* = 3/group). **j**, **k**. Construction of OE-EMP1, sh-EMP1 by plasmid transfection cell lines, HSC activation was induced by administration of TGF-β, and it was detected whether the expression of EMP1 interfered with HSC activation at the mRNA (*n* = 5/group) and protein (*n* = 3/group) levels. All data used for statistics above are SD ± mean, n.s > 0.05, * *P* < 0.05, ** *P* < 0.01, *** *P* < 0.001, and were analyzed by two-way ANOVA or t-test followed by Tukey test

To clarify the function of EMP1 and its role in MASLD-IRI pathogenesis, we used the UCSC CellBrowser single-cell sequencing database for bioinformatic analysis, and found that EMP1 is mainly expressed in liver non-parenchymal cells, including liver sinusoidal endothelial cells (LSECs), HSCs, and kupffer cells (KCs), but is mostly absent in hepatocytes (Fig. 3a,b). By employing an OA/PA (oleic acid/palmitic acid) + IRI cellular model of MASLD-IRI, we everified that EMP1 was predominantly enriched in LSECs and HSCs (Fig. 3c,d). we then further evaluated the expression levels of EMP1 in different cells under single injury (OA/PA or IRI) and compound injury (OA/PA + IRI) conditions. The results showed that EMP1 expression was further upregulated by the combined injury condition compared to the single injury group only in LSECs and HSCs; therefore, we did not evaluate KCs for further analyses (Fig. 3e,f). Spatial transcriptomic analysis has revealed that liver lobules are typically divided into four zones (Zone 1–Zone 4) [26, 27]. EMP1 expression was primarily concentrated in Zone 2 and Zone 3 areas near the central vein (Fig. S4a), and its distribution overlapped with that of LSECs and HSCs.

We first investigated LSECs, which showed the highest enrichment of EMP1. LSECs are involved in liver inflammation and fibrosis mainly through the loss of fenestration and capillarization, disrupting intercellular communication [28, 29]. However, EMP1 overexpression in LSECs did not exacerbate capillarization or enhance the secretion of TGF-β, a key activator of HSCs (Fig. S5a-f). We subsequently focused on EMP1 in HSCs. Immunofluorescence staining showed that EMP1 co-localized with α-SMA and was simultaneously upregulated in the HFD-LT group (Fig. 3g). Analysis via GEPIA2 revealed that EMP1 expression was highly- to moderately-positively correlated with established HSC activation markers including ACTA2 (α-SMA),COL1A2, and TGF-β, and was also moderately positively correlated with inflammatory mediators of HSC activation such as IL6 (Fig. 3h). Therefore, EMP1 in HSCs could promote their autonomous activation.

OA/PA + IRI co-stimulation significantly increased TGF-β secretion by HSCs, reaching levels comparable to those observed with direct treatment with 10 ng/mL TGF-β. Both stimulation methods showed similar upregulation of EMP1 and HSC activation markers (Fig. S6a-d). Therefore, in subsequent cellular experiments, we used TGF-β to directly induce HSC activation. To investigate the functional role of EMP1, we established control, EMP1-overexpressing (OE-EMP1), and EMP1-knockdown (sh-EMP1) cell lines (Fig. S6e–h). EMP1 overexpression enhanced HSC activation at both protein and transcript levels, while EMP1 knockdown inhibited HSC activation, which was further verified by immunofluorescence (Fig. 3i-k).

Collectively, these results indicate that while EMP1 is present in multiple non-parenchymal cell types, its expression in HSCs specifically governs their activation. We therefore propose that EMP1 upregulation represents a critical driver of HSC activation and inflammatory liver injury during MASLD-IRI, although the underlying molecular mechanisms require further elucidation.

### EMP1 promotes FAK phosphorylation leading to HSC activation

**Fig.4:**
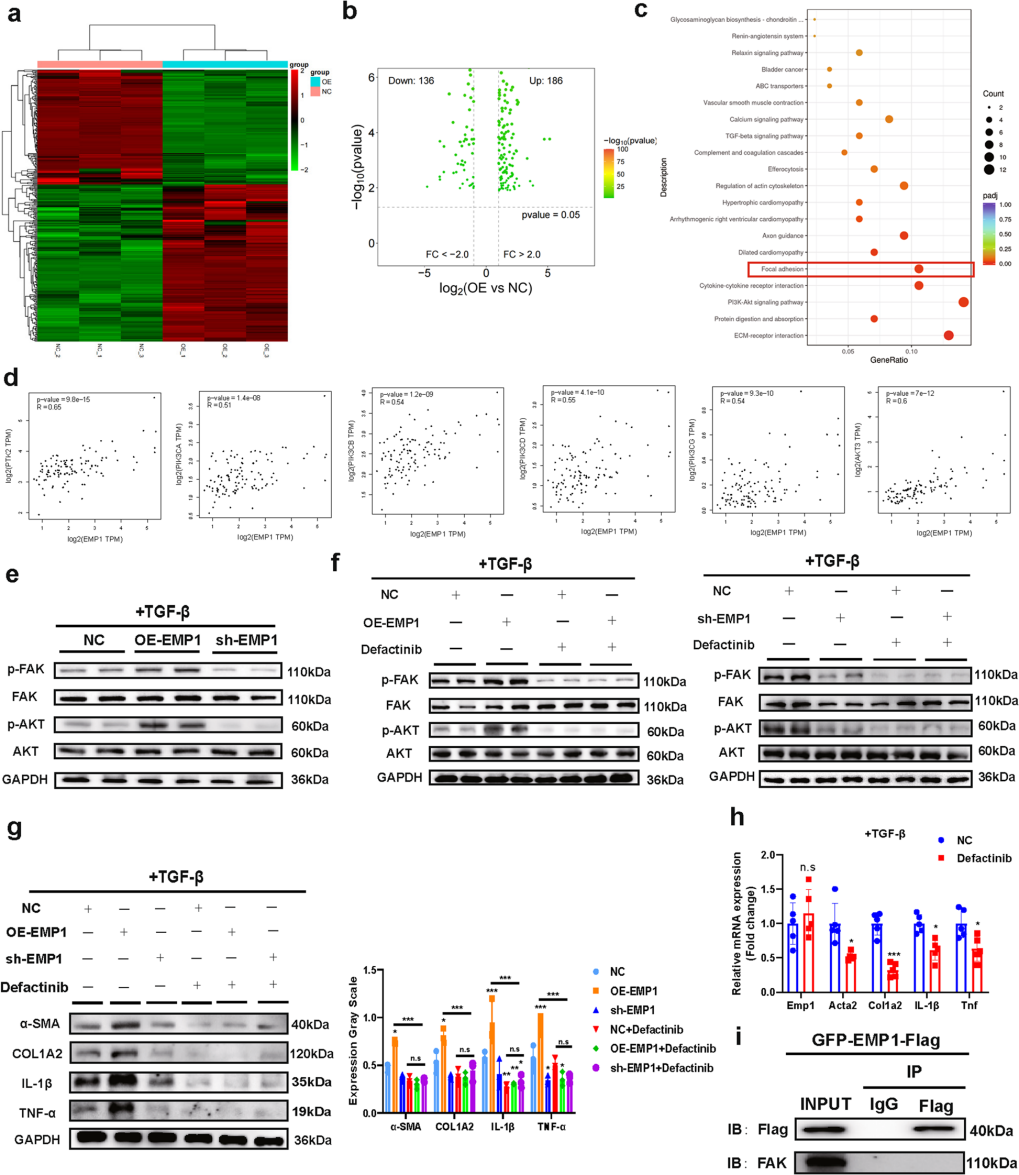
EMP1 mediates HSC activation indirectly through FAK phosphorylation. **a**, **b**. Using NC and OE-EMP1 cell lines again transcriptome sequencing (*n* = 3/group), heatmaps and volcano plots showed significant differences. **c**. Enrichment analysis of the differential genes based on the KEGG database was performed and shown in bubble plots, again enrichment to FA signaling pathway genes with a large number of significant differences. **d**. Correlation of EMP1 expression in liver with key genes in FA signaling pathway, such as PTK2, PIK3C, AKT3, etc., was analyzed by using GEPIA2. **e**. Detection of the role of EMP1 expression modulation in FAK and its downstream AKTs in the FA signaling pathway in HSC cell lines under TGF-β-induced activation (*n* = 4/group). **f**. Under the intervention of FAK phosphorylation inhibitor Defactinib intervention, the effect of EMP1 expression modulation on FAK-AKT phosphorylated protein levels was again examined (*n* = 4/group). **g**. Detection of HSC activation and inflammatory protein expression under OE-EMP1, sh-EMP1 or FAK phosphorylation inhibition intervention (*n* = 3/group). **h**. Detection of HSC activation and mRNA expression of inflammatory markers under FAK phosphorylation inhibition (*n* = 5/group). **i**. CoIP assay of EMP1 with FAK by constructing GFP-EMP1-Flag cell line. All data used for statistics above are SD ± mean, n.s > 0.05, * *P* < 0.05, ** *P* < 0.01, *** *P* < 0.001, and were analyzed by two-way ANOVA or t-test followed by Tukey test

To investigate the mechanisms underlying the activation of HSCs by EMP1, we conducted RNA-seq in OE-EMP1 and normal control (NC) cells. Heatmap and volcano plot analyses revealed substantial transcriptomic alterations upon EMP1 modulation (Fig. 4a,b). KEGG enrichment analysis of DEGs indicated significant enrichment of the FA signaling pathway, consistent with prior tissue sequencing findings (Fig. 4c; Fig. 2d), implicating this pathway in MASLD-IRI and HSC activation. Using GEPIA2, we assessed the correlation between EMP1 expression and key components of the FA pathway, including PTK2 (FAK), various subunits of PIK3C (PI3K), and AKT3. EMP1 expression exhibited moderate positive correlations with these genes (Fig. 4d). Previous studies have demonstrated that the FA signaling pathway promotes HSC activation by activating the PI3K/AKT pathway through FAK phosphorylation [30, 31]. We therefore investigated whether the effect of EMP1 on HSC activation is mediated via the FAK/PI3K/AKT signaling axis.

Protein quantification assays showed that neither overexpression nor knockdown of EMP1 markedly affected total FAK expression. However, EMP1 upregulation significantly enhanced FAK and AKT phosphorylation, while EMP1 silencing suppressed it (Fig. 4e). Treatment with the FAK phosphorylation inhibitor defactinib abolished EMP1-mediated effects on FAK and AKT phosphorylation (Fig. 4f) and mitigated HSC activation (Fig. 4g). Moreover, defactinib alone suppressed HSC activation and inflammatory responses without affecting EMP1 expression (Fig. 4h), indicating that EMP1 acts upstream of FAK and depends on FAK phosphorylation to regulate HSC activation.

Although these results suggest that EMP1 promotes FAK phosphorylation, co-immunoprecipitation assays revealed no direct interaction between EMP1 and FAK (Fig. 4i), suggesting that EMP1 regulates FAK phosphorylation indirectly, potentially through an intermediate molecule. Further studies are warranted to clarify this regulatory axis.

### Regulation of FAK phosphorylation by EMP1 is dependent on TLN1

**Fig.5:**
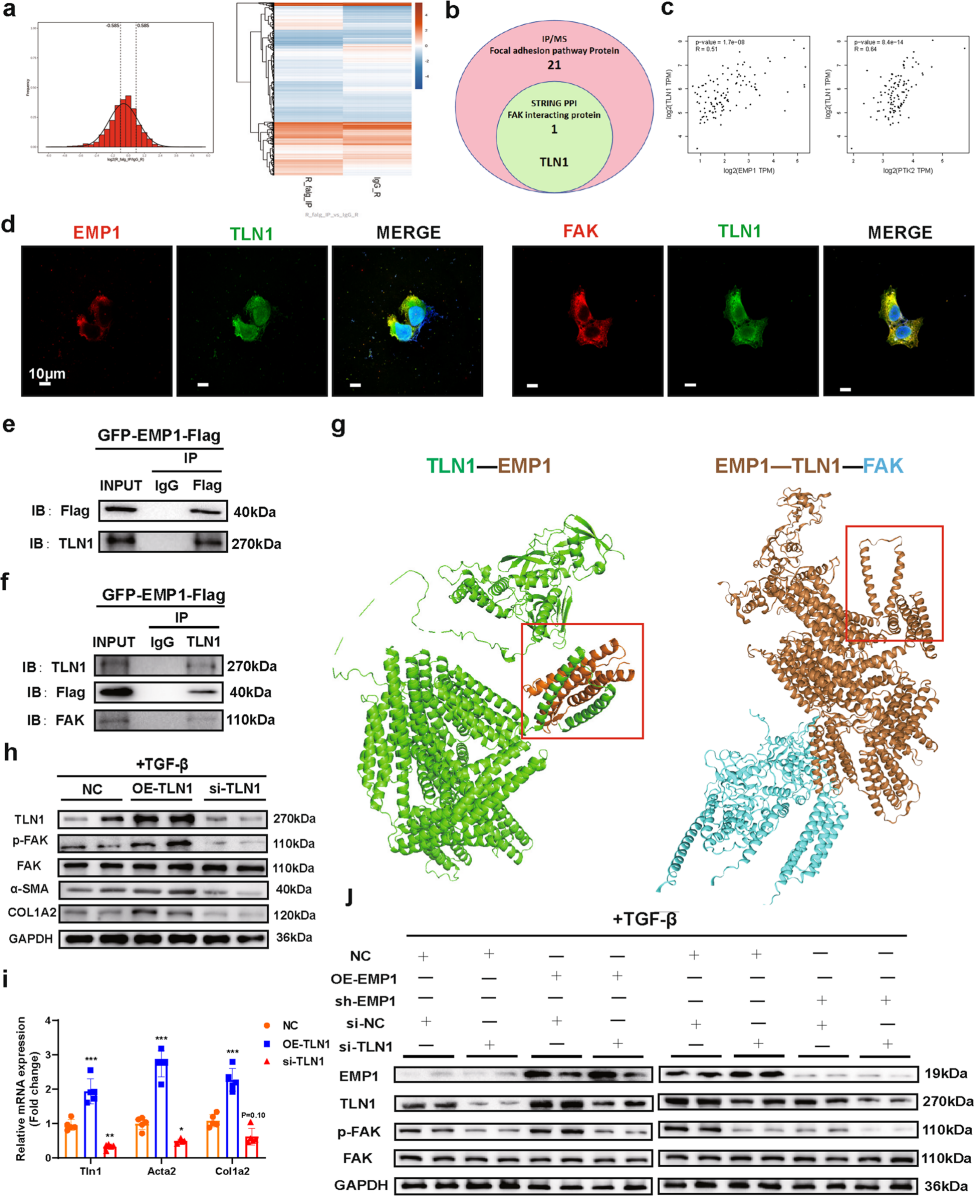
Regulation of FAK phosphorylation by EMP1 is dependent on the role of TLN1. **a**. CoIP/MS was performed using NC and OE-EMP1 cell lines to identify possible EMP1-interacting proteins by proteomics. **b**. Combining the CoIP/MS data with the STRING protein-interaction database to analyze the proteins that can proteinally interact with both EMP1 and FAK, TLN1. **c**. GEPIA2 analysis of the correlation between TLN1 expression and EMP1 and FAK in liver. **d**. Immunofluorescence staining of EMP1, TLN1, and FAK followed by confocal photography (*n* = 3/group) was used to detect the intracellular co-localization between EMP1/TLN1 and TLN1/FAK. The scale bar = 10 μm. **e**, **f**. Constructed GFP-EMP1-Flag cell-stable transfections and verified EMP1/TLN1/FAK interactions by CoIP. **g**. Simulated EMP1/TLN1 and TLN1/FAK docking models using Alphafold3 and HDOCK to analyze the structural domains, and the red boxes are EMP/TLN1 docking structural domains. **h**. Detection of mRNA expression of activation markers of HSC under the regulation of TLN1 expression in HSC(*n* = 5/group). **i**. FAK and its phosphorylation levels were examined under the regulation of TLN1 expression in HSC, and protein expression of HSC activation markers (*n* = 4/group). **j**. Used the OE-EMP1, sh-EMP1 cell lines transfected with si-NC or si-TLN1, respectively, and detected whether FAK's protein phosphorylation level is co-influenced by EMP1, TLN1 (n = 4/group)

To identify direct binding targets of EMP1 in HSCs and investigate potential intermediaries in the EMP1–FAK axis, we performed co-immunoprecipitation coupled with mass spectrometry (Co-IP/MS) in NC and OE-EMP1 cells. Following peptide quantification, cleavage site analysis, and protein quantification, we identified numerous significantly altered proteins (Fig. 5a; Fig. S7a–d). KEGG enrichment analysis revealed 21 proteins from the FA pathway with potential interactions with EMP1. Mining of the STRING protein interaction database (Fig. S7e) indicated that, among these, only TLN1 was predicted to directly bind EMP1 and FAK (Fig. 5b), suggesting that TLN1 may mediate EMP1-dependent regulation of FAK.

TLN1 is a cytoskeletal protein involved in cell adhesion, migration, and mechanosignaling [32]. Previous studies have shown that TLN1 recruits FAK to adhesion sites via binding between its N-terminal FERM and the FAK FAT domains, promoting FAK autophosphorylation and subsequent activation of downstream pathways including the PI3K/AKT and Ras/MAPK pathways, thereby influencing cell proliferation, survival, and migration [33, 34]. Using GEPIA2, we further confirmed that TLN1 expression in liver tissue significantly correlated with EMP1 and FAK (PTK2) (Fig. 5c). These findings support the hypothesis that an EMP1/TLN1/FAK complex may mediate HSC activation, though functional validation is required.

Next, we first performed immunofluorescence and confocal imaging, revealing that EMP1 was primarily localized to the plasma membrane and cytoplasm. TLN1 was ubiquitously expressed, and FAK was primarily localized in the plasma membrane and cytoplasm, with minimal nuclear presence. Importantly, TLN1 co-localized with EMP1 and FAK in the membrane and cytoplasm (Fig. 5d). Subsequent Co-IP experiments confirmed interactions between TLN1 and EMP1, as well as between TLN1 and FAK (Fig. 5e, f), supporting the existence of an EMP1/TLN1/FAK complex, particularly at the membrane. Structural modeling with AlphaFold3 and HDOCK suggested stable docking among the three proteins, with EMP1 and FAK binding to distinct domains of TLN1 (Fig. 5g). These data indicate that the complex likely exists, but further studies are needed to verify TLN1-dependent regulation of FAK phosphorylation by EMP1.

We then overexpressed TLN1 in HSCs and found that under TGF-β-induced activation, TLN1 upregulation significantly enhanced FAK phosphorylation and promoted expression of HSC activation markers compared to those in controls (Fig. 5 h, i). To determine whether TLN1 mediates EMP1-dependent FAK phosphorylation, we transfected EMP1-overexpressing cells with TLN1 siRNA. EMP1 upregulation increased TLN1 expression and promoted FAK phosphorylation, whereas TLN1 knockdown markedly attenuated this effect (Fig. 5 j). These results demonstrate that EMP1 regulates FAK phosphorylation in a TLN1-dependent manner. However, because EMP1 is not a transcription factor, the precise mechanism by which it binds to TLN1 and enhances TLN1 expression remains unclear.

### EMP1 competes for the SMURF1 binding site to inhibit ubiquitination-mediated degradation of TLN1

**Fig.6:**
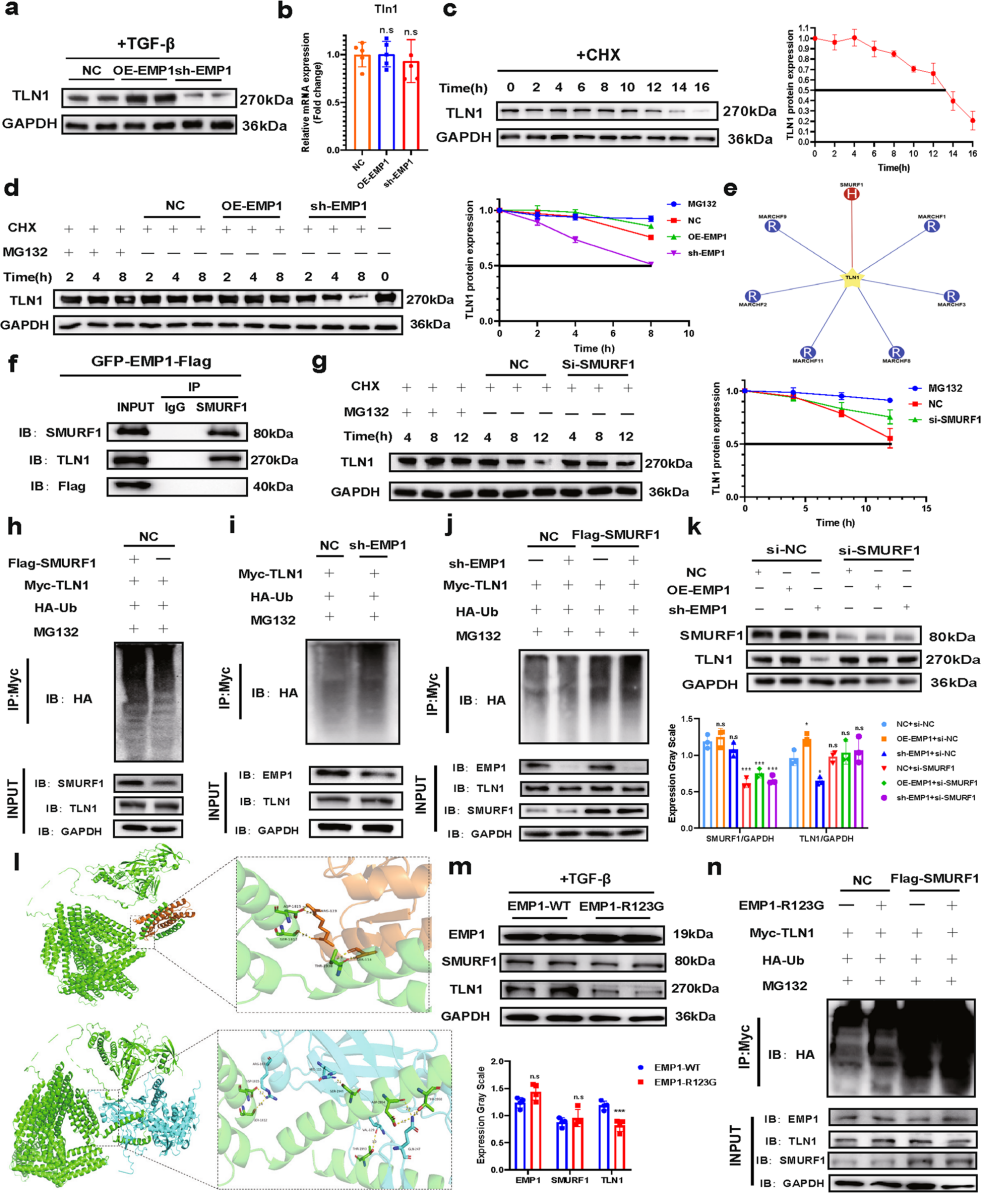
EMP1 inhibits TLN1 degradation by ubiquitination of SMURF1 by competing for binding residues. **a**. WB experiments were performed to detect changes in TLN1 expression (*n* = 4/group) in different cell lines. **b**. qRT-PCR to detect the TLN1 mRNA levels (*n* = 5/group) under different EMP1 expression scenarios. **c**. protein half-life of TLN1 under CHX intervention (*n* = 3/group); **d**. TLN1 protein expression levels (*n* = 3/group) were examined under different EMP1 scenarios at 2, 4, and 8 h under CHX inhibition of neo-protein production to assess TLN1 degradation.** e**. Analysis of E3 ubiquitin ligases that can bind to TLN1 using UbiBrowser 2.0. **f**. Validation of the relationship of SMURF1 to TLN1 and EMP1 binding, respectively. **g**. Degradation of TLN1 protein by UPS at 4, 8, and 12 h of CHX induction in MG132, NC, and si-SMURF1 groups, respectively (*n* = 3/group). **h**. TLN1 ubiquitination levels were assayed in the NC group transfected with Myc-TLN1, HA-Ub, and given MG132 to inhibit proteasomal degradation with or without Flag-SMURF1. **i**. The NC and sh-EMP1 groups were transfected with Myc-TLN1, HA-Ub, and given MG132 to inhibit proteasomal degradation, the ubiquitination level of TLN1 was assayed. **j**. The ubiquitination level of TLN1 was assayed by giving NC and sh-EMP1 transfected with Myc-TLN1, HA-Ub, and given MG132 to inhibit proteasomal degradation, with or without Flag-SMURF1. **k**. Altered protein expression of SMURF1 and TLN1 after transfection of NC, OE-EMP1, and sh-EMP1 cell lines with si-NC or si-SMURF1, respectively (*n* = 3/group). **l**. Using AlphaFold3 and HDOCK to predict the most likely binding residues and protein models for EMP1/TLN1 and SMURF1/TLN1. **m**. EMP1, SMURF1, and TLN1 protein expression assays in EMP1-WT cell line and EMP1-R123G mutant cell line (*n* = 4/group). **n**. TLN1 ubiquitination assay in EMP1-R123G mutant cell lines with and without SMURF1 overexpression. All data used for statistics above are SD ± mean, n.s > 0.05, * *P* < 0.05, ** *P* < 0.01, *** *P* < 0.001, and were analyzed by two-way ANOVA or t-test followed by Tukey test

To investigate how EMP1 regulates TLN1 expression, we assessed protein and transcript levels of TLN1 following modulation of EMP1 expression. EMP1 perturbation affected TLN1 protein but not mRNA expression (Fig. 6a,b), suggesting that EMP1 regulates TLN1 through post-translational modifications. The concordance in their expression patterns further implies that EMP1 may protect TLN1 from protein degradation. Given the major role of the ubiquitin–proteasome system (UPS) in targeted protein degradation, specifically through E3 ubiquitin ligase-mediated ubiquitination and subsequent proteasomal recognition [35], we hypothesized that EMP1 might inhibit UPS recognition of TLN1.

To further investigate whether EMP1 inhibits TLN1 degradation, we first blocked de novo protein synthesis using cycloheximide (CHX) and determined that the half-life of TLN1 is approximately 12–14 h (Fig. 6c). Subsequent experiments revealed that OE-EMP1 enhanced TLN1 stability under CHX treatment, whereas sh-EMP1 group led to a decrease in the half-life of TLN1 to approximately 8 h. (Fig. 6d). Previous studies have indicated that the stability of TLN1 is tightly regulated by ubiquitination [36, 37]. Therefore, we propose that EMP1 upregulation may inhibit TLN1 degradation specifically by interfering with its ubiquitination, although more direct evidence is required to validate this mechanism.

To identify relevant E3 ubiquitin ligases, we analyzed potential TLN1-interacting enzymes using UbiBrowser 2.0 and identified SMURF1 as the top candidate (Fig. 6e), consistent with previous reports demonstrating that SMURF1, a HECT-type E3 ligase, ubiquitinates TLN1 by recognizing its FERM domain [38]. Although other E3 ligases may also interact with TLN1 (Fig. S8a,b), SMURF1 is of particular interest due to its established roles in TGF-β and Hippo signaling—pathways critically involved in MASLD, liver fibrosis, and HCC [39–41]. Therefore, we further investigated the regulation of TLN1 by SMURF1. Co-IP confirmed that SMURF1 binds to TLN1 (Fig. 6f). Moreover, silencing SMURF1 significantly inhibited the ubiquitination of TLN1 at 12 h (Fig. 6g). These findings suggest that SMURF1 promotes TLN1 ubiquitination and degradation, though whether EMP1 interferes with this process remains to be determined.

Subsequently, we generated a series of cell lines that were transfected in different combinations with NC and sh-EMP1, together with plasmids encoding Flag-SMURF1, Myc-TLN1, and HA-ub. MG132 was used to inhibit proteasomal degradation, allowing us to assess how modulation of EMP1 and SMURF1 expression affected TLN1 ubiquitination. Evaluation of the effect of SMURF1 overexpression on TLN1 ubiquitination in NC cell lines revealed that TLN1 ubiquitination was significantly elevated in the Flag-SMURF1-transfected group compared with that in the NC group (Fig. 6h). In the NC background, transfection of sh-EMP1 together with HA-Ub and Myc-TLN1, followed by MG132 treatment, revealed that knockdown of EMP1 significantly increased TLN1 ubiquitination (Fig. 6i). We further combined the interventions of EMP1 and SMURF1 and re-examined TLN1 ubiquitination. The results confirmed that SMURF1 overexpression increased the ubiquitin-binding capacity of TLN1, while EMP1 deletion further enhanced TLN1 ubiquitination in the presence of SMURF1 (Fig. 6j), indicating that EMP1 has an inhibitory effect on SMURF1 binding to TLN1 for ubiquitination. Following SMURF1 knockdown, modulation of EMP1 expression no longer influenced TLN1 protein levels (Fig. 6k), suggesting that EMP1 regulates TLN1 in a SMURF1-dependent manner, though the exact mechanism has yet to be defined.

Protein conformational analysis revealed that SMURF1 and EMP1 bind to the C-terminal structural domain of TLN1, and both directly compete for binding to the ASP1815 site (Fig. 6l), suggesting that EMP1 competes with SMURF1 for binding to the same amino acid residues of TLN1. We thus hypothesized that elevated EMP1 in the MASLD-IRI environment promotes its binding to the TLN1-D1815 site via EMP1-R123, preventing SMURF1 association and subsequent TLN1 degradation through the UPS. To test this hypothesis, we constructed an EMP1 p.R123G (EMP1-R123G) gene point mutant cell line, which abolishes the ability of EMP1 to interact with TLN1 at the ARG123 site. The results showed that the EMP1-R123G cell line was significantly less resistant to TLN1 degradation compared to the WT line in the presence of HSC activation (Fig. 6m). A ubiquitination assay further confirmed that EMP1-R123G lost the ability to compete with SMURF1 for binding to TLN1, thereby failing to prevent TLN1 ubiquitination (Fig. 6n). These findings confirm that EMP1 inhibits TLN1 ubiquitination by competitively blocking SMURF1 binding.

Further, we examined SMURF1 expression and its association with the EMP1/TLN1/FAK pathway in animal and cellular models. Unexpectedly, we found that MASLD-IRI upregulated SMURF1 expression in both animal and cellular models, but this upregulation did not inhibit TLN1 expression (Fig. S8a-d). To clarify this observation, we repeated the experiments in EMP1-silenced animal and cellular models, As expected, MASLD-IRI–mediated up-regulation of SMURF1 expression after EMP1 silencing successfully suppressed TLN1 expression (Fig. S8e), while modulation of EMP1 expression alone did not affect SMURF1 levels (Fig. S8f-h). These findings further confirm that EMP1 up-regulation inhibits SMURF1-mediated ubiquitination of TLN1.

### EMP1 silencing attenuates HSC cactivation-induced liver injury via TLN1/FAK axis

**Fig.7:**
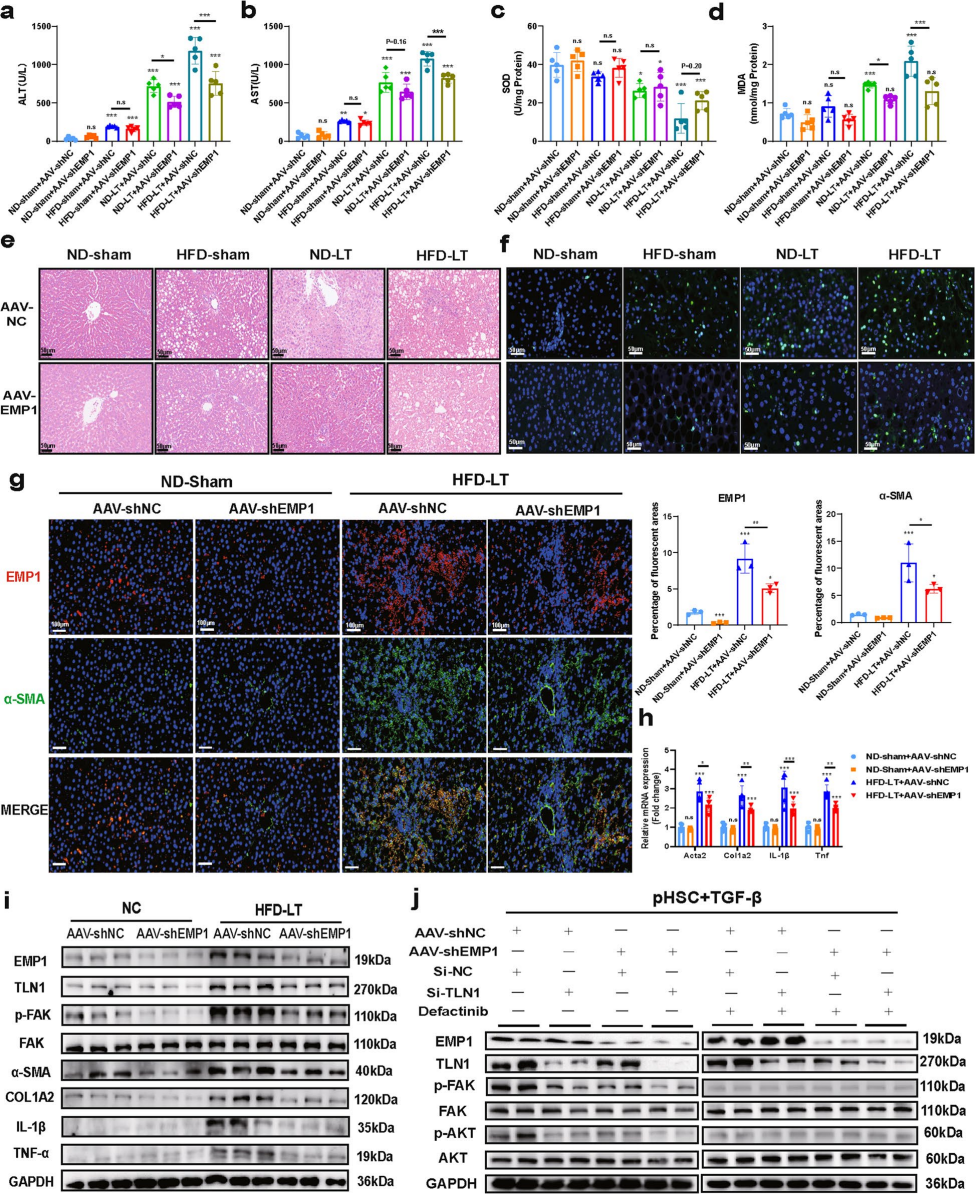
EMP1 selective silencing inhibition attenuates MASLD-IRI-induced liver injury by inhibiting HSC activation via TLN1/FAK. Figure 7 The same 4 groups of animal model treatments were performed as in Fig. 1. The difference is that AAV-shNC and AAV-shEMP1 injections were given to the donors in each group separately, and the area was divided into 8 groups. **a**, **b**. Detection of liver injury markers ALT and AST in serum to check the overall level of liver injury (*n* = 5/group). **c**, **d**. Detection of oxidative products SOD and MDA in liver to reveal the level of oxidative stress-related injury (*n* = 5/group). **e**, **f**. Pathological examination of rat liver tissues to observe the type of liver injury, H&E showed tissue edema, structural disorders, and necrosis in liver. Tunel staining demonstrated the degree of liver cell death in different models, both scale bar = 50 μm(n = 3/group). **g**. By double immunofluorescence staining of liver tissues with EMP1 and α-SMA, it was observed that EMP1 co-localized with activated HSC, and there was a common up-regulation of the expression in terms of the expression amount. Where red is EMP1, green is α-SMA, and blue is DAPI cell nuclei. The scale bar = 100 μm (*n* = 3/group). **h**. Detection of mRNA expression of HSC activation, and level of inflammation in ND-sham and HFD-LT hepatic tissues after EMP1-specific silencing (n = 5/group). **i.** Detection of protein expression (*n* = 3/group) of EMP1/TLN1/FAK phosphorylation, degree of HSC activation, and level of inflammation after EMP1-specific silencing. **j**. AAV-shNC, AAV-shEMP1 primary hepatic stellate cells were extracted and grouped by whether or not they were transfected with si-TLN1 and whether or not they were given a FAK phosphorylation inhibitor, and TGF-β was given to stimulate hepatic stellate cell activation, and the effect of EMP1 or TLN1 silencing on the phosphorylation levels of the EMP1/TLN1/FAK/AKT proteins was examined (*n* = 4/group). All data used for statistics above are SD ± mean, n.s > 0.05, * *P* < 0.05, ** *P* < 0.01, *** *P* < 0.001, and were analyzed by two-way ANOVA or t-test followed by Tukey test

To verify whether EMP1 promotes HSC activation to aggravate MASLD-IRI-induced liver injury through the TLN1/FAK signaling axis, we established EMP1-silenced animal models via tail vein injection of AAV6-shRNA, selected for its efficient targeting of HSCs [25, 42, 43]. The animals were divided into ND-Sham, HFD-Sham, ND-LT, and HFD-LT groups. The results demonstrated that although EMP1 silencing tended to mitigate liver injury across all experimental groups, a statistically significant reduction in ALT and AST levels was observed exclusively in the HFD-LT group (Fig. 7a,b). Similarly, EMP1 knockdown attenuated oxidative stress in MASLD-IRI, as indicated by a marked decrease in the lipid peroxidation marker MDA; however, the antioxidant factor SOD showed only a non-significant increasing trend (Fig. 7c,d). Further histopathological evaluation via H&E and TUNEL staining confirmed that EMP1 silencing alleviated hepatic structural damage and reduced cell death specifically in the HFD-LT group (Fig. 7e,f). These findings imply a potential therapeutic role for EMP1 targeting in MASLD-IRI, whereas its effects in other groups remained minimal.

We next assessed whether EMP1-specific silencing in vivo influenced HSC activation. Consistent with the in vitro findings, EMP1 knockdown significantly reduced the fluorescence intensity of α-SMA (Fig. 7g). Given that HSC activation promotes liver fibrosis and MASLD-IRI enhances fibrosis susceptibility without causing immediate fibrosis (Fig. S2), we next assessed whether EMP1 silencing mitigates fibrosis in a CCl₄-induced model. EMP1 knockdown reduced CCl₄-induced liver injury, with a non-significant decrease in γ-glutamyl transpeptidase (GGT) (Fig. S10a-d). ELISA showed elevated ECM1 and reduced hyaluronic acid levels (Fig. S10e,f). Pathological staining confirmed reduced injury and collagen deposition (Fig. S10g,h), supported by corresponding changes in ECM1 and CD44 expression at protein and mRNA levels (Fig. S10i,j). These findings indicate that EMP1-mediated HSC activation contributes to injury across acute and chronic liver models.

We then validated the involvement of the EMP1/TLN1/FAK axis. EMP1 knockdown downregulated TLN1, reduced FAK and AKT phosphorylation, and decreased expression of α-SMA, COL1A2, IL-1β, and TNF-α at protein and mRNA levels (Fig. 7h,i), consistent with the in vitro results, confirming that EMP1 silencing attenuates HSC activation and inflammatory responses via the TLN1/FAK axis, thereby mitigating MASLD-IR injury. For further verification, we isolated primary HSCs from AAV-shNC and AAV-shEMP1 groups, and activated them with TGF-β, with further treatment with either si-TLN1 or defactinib. Disruption of any component within the EMP1/TLN1/FAK axis suppressed AKT phosphorylation and downstream signaling (Fig. 7j), highlighting the essential and non-redundant role of each component in HSC activation.

Collectively, these findings demonstrate that the EMP1/TLN1/FAK pathway significantly regulates HSC activation across diverse liver injury contexts.

### Prognostic monitoring value of EMP1 in MASLD-IRI

**Fig.8:**
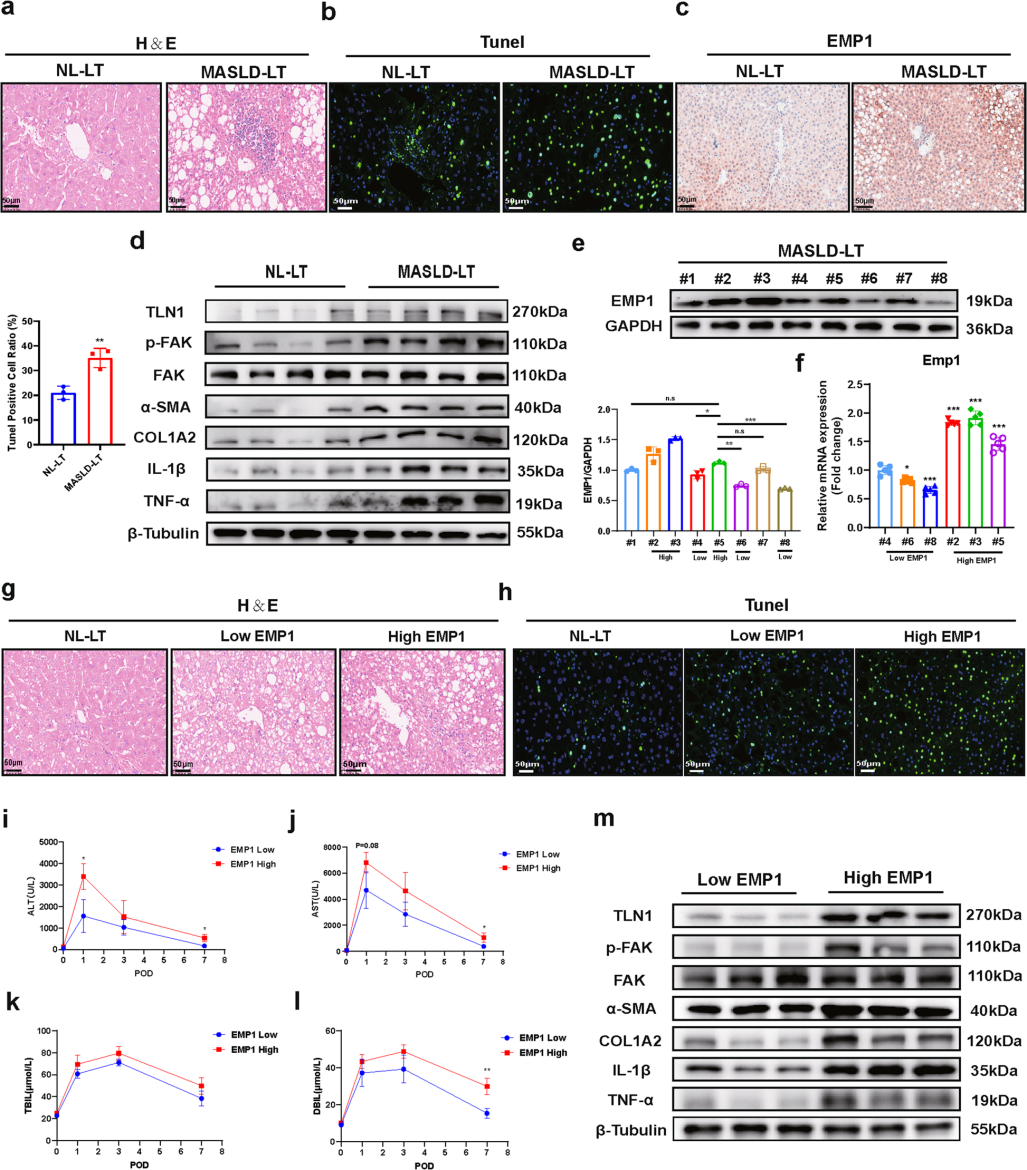
Validation of EMP1 as a prognostic monitoring marker for clinical MASLD donor liver transplantation. NL-LT: group of patients who underwent normal liver transplantation; MASLD-LT: group of patients who underwent MASLD donor transplantation.Low EMP1: group of patients with MASLD-LT with relatively low expression of EMP1; High EMP1: group of patients with MASLD-LT with relatively high expression of EMP1. **a-c**. Pathological examination of NL-LT and MASLD-LT human liver tissues to observe the type of liver injury, H&E showed tissue edema, structural disorders, and necrosis in liver.scale bar = 50 μm (*n* = 3/group).Tunel staining demonstrated the degree of liver cell death in different models, scale bar = 50 μm(*n* = 3/group). Immunohistochemical staining of EMP1, Scale bar = 50 μm (*n* = 3/group). **d**. Protein expression assays of the TLN1/FAK axis, activation markers of HSC, and inflammatory factors were performed on the NL-LT and MASLD-LT groups (*n* = 4/group). **e**, **f**. The samples were divided into two groups, EMP1 Low and EMP1 High, by detecting EMP1 protein expression (*n* = 3/group) in eight samples of human tissue samples from the MASLD-LT group, And it was verified by mRNA detection (*n* = 5/group). **g**, **h** Pathological examination of NL-LT, EMP1 Low and EMP1 High human liver tissues to observe the type of liver injury, H&E showed tissue edema, structural disorders, and necrosis in liver. scale bar = 50 μm (*n* = 3/group).Tunel staining demonstrated the degree of liver cell death in different models, scale bar = 50 μm (*n* = 3/group). **i-l**. Clinical data of ALT,AST,TBIL and DBIL at postoperative days 0,1,3 and 7 were counted for both EMP1 Low and EMP1 High groups (*n* = 3/group). **m**. TLN1/FAK axis, HSC activation markers, and inflammatory factor protein expression were verified by human liver samples in both EMP1 Low and EMP1 High groups (*n* = 3/group)

We collected 18 liver transplantation samples and corresponding clinical data from Wuhan Union Hospital, including eight from MASLD donors. Baseline donor and recipient characteristics are summarized in Supplementary Tables 1 and 2. We confirmed that MASLD donor livers exhibited more severe structural disruption and cell death (Fig. 8a,b), along with significant upregulation of EMP1 expression using immunohistochemistry (Fig. 8c). Consistent with our animal and cellular findings, EMP1 upregulation was accompanied with increased levels of TLN1, HSC activation markers, and inflammatory factors (Fig. 8d).

The eight MASLD donor samples were further stratified based on EMP1 protein expression. Samples #2, #3, and #5, with the highest EMP1 levels, were designated as the EMP1-High group; samples #4, #6, and #8, which were statistically different from #5, comprised the EMP1-Low group. Analysis of transcript levels confirmed this grouping (Fig. 8e,f).

Comparison with normal donor livers (NL-LT) revealed that NL-LT and EMP1-Low groups showed less histological disorganization and cell death than that observed in the EMP1-High group (Fig. 8g,h). Analysis of postoperative clinical indicators showed that the EMP1-High group had higher ALT levels on day 1, and significantly elevated ALT, AST, and DBIL on day 7 compared to those in the EMP1-Low group (Fig. 8i–l), suggesting more severe and prolonged liver injury. Additionally, the EMP1-High group exhibited higher TLN1 expression, FAK phosphorylation, HSC activation, and inflammatory responses (Fig. 8m), further validating that the EMP1/TLN1/FAK axis promotes HSC activation and inflammatory factor release.

## Discussion

In this study, we demonstrated that the use of MASLD donor livers exacerbates transplantation-associated IRI. This aggravated injury was closely associated with HSC hyperactivation and subsequent inflammatory factor release. Sequencing analyses and experimental validation revealed a significant upregulation of EMP1 under MASLD-IRI conditions. Subsequent analyses revealed that EMP1 upregulation promoted HSC activation and release of inflammatory factors, highlighting its role as a key mediator in MASLD-IRI–induced liver injury.

Despite alleviating organ shortages, the use of MASLD donor livers poses a major clinical challenge due to their heightened susceptibility to postoperative graft dysfunction [1, 44]. Current perioperative strategies—including donor metabolic optimization, mechanical perfusion with antioxidants, and dynamic postoperative monitoring—mainly target general IRI mechanisms such as oxidative stress and cell death [45], rather than MASLD-specific injury pathways. Consistent with clinical observations and recent evidence [46–49], we found that MASLD donor livers are associated with adverse outcomes including nonfunction, delayed recovery, and acute rejection, culminating in transplant failure and reduced long-term survival. This susceptibility to IRI in steatotic livers has been attributed to immune dysregulation, metabolic disturbances, gut microbiota-mediated inflammation, oxidative stress, and cell death [44–50]. Our findings highlight HSC activation as a central event linking inflammation and fibrosis in liver diseases such as MASLD and IRI [6–11]. However, the specific molecular mechanisms underlying this process in MASLD-IRI are not well understood. We therefore investigated whether a specific target could be identified to modulate HSC activation, thereby providing new avenues for diagnosis and therapy in MASLD donor liver transplantation.

Combining sequencing, biosignature analysis, and molecular biology experimental validation, we identified EMP1 as a potential target. Current studies on EMP1 mainly focus on tumor invasion, with its molecular function remaining controversial. A recent study reported that EMP1 could be used as a positive target for liver metastasis in colorectal cancer [13]. However, EMP1 has been reported as an oncogene in gastric cancer [51]. Moreover, the roles of EMP1 in the liver are poorly reported, and it is currently only predicted as a potential marker for liver fibrosis and hepatocellular carcinoma by bioinformatics analysis [15, 52]. Therefore, further studies are required to clarify the roles of EMP1 in the context of liver and non-neoplastic diseases.

Our findings demonstrate that EMP1upregulation in MASLD-IRI promotes HSC activation through the TLN1/FAK signaling axis. Specifically, EMP1 upregulation stabilizes TLN1 expression by inhibiting its ubiquitin-mediated degradation. Because EMP1 and SMURF1 share the same C-terminal binding site on TLN1, increased EMP1 competitively prevents SMURF1–TLN1 association and subsequent ubiquitination of TLN1. Accumulated TLN1 then promotes FAK phosphorylation through direct interaction via its N-terminal FERM domain [38, 53], which in turn drives HSC activation through downstream pathways including PI3K-AKT and Ras-MAPK [30, 54]. Although SMURF1 expression was upregulated in MASLD and IRI and has been reported to protect against renal IRI [55], we observed that its ability to promote TLN1 degradation in the liver was only evident when EMP1 was silenced, possibly reflecting tissue-specific differences in the expression or interaction of EMP1. Further analysis revealed that blocking any component of EMP1/TLN1/FAK significantly inhibited HSC activation, further validating this mechanism.

Our study identifies the EMP1/TLN1/FAK axis as an amplification mechanism underlying liver injury in MASLD-IRI and proposes novel clinical strategies. For instance, pre-transplant targeting of EMP1 via silencing or small-molecule inhibitors could suppress HSC-driven inflammation and mitigate fibrotic susceptibility in MASLD grafts. However, clinical translation requires addressing several challenges including refining organ-specific delivery systems to avoid off-target effects, validating the correlation between EMP1 expression and postoperative recovery in clinical cohorts to establish EMP1-based donor risk evaluation, and integrating EMP1-targeting approaches with existing interventions (e.g., ischemic preconditioning) in combinatorial regimens. Notably, emerging epigenetic therapies such as histone deacetylase inhibitors [56, 57] may offer alternative strategies for modulating EMP1 expression and improving graft quality.

Despite these findings, some limitations should be acknowledged. First, although transcriptomic analysis of our disease model revealed significant upregulation of expression of Spp1 and Itga3 alongside Emp1, their functional roles were not thoroughly investigated. Previous studies indicate that macrophage-derived SPP1 impairs fatty acid oxidation in MASH via STAT3 signaling, and suppression of Spp1 by C/EBPα markedly reduces inflammatory fibrogenesis in MASH [58, 59]. Additionally, Itga3 has been shown to promote glycolysis through FAK phosphorylation in pancreatic cancer [60], suggesting that both genes may also significantly contribute to injury in MASLD donor liver transplantation and warrant further exploration. Furthermore, after TLN1 silencing, the changes in FAK phosphorylation across the NC, OE-EMP1, and Sh-EMP1 groups were not fully consistent, implying that EMP1 may regulate FAK through TLN1-dependent and independent mechanisms. Future studies should focus on elucidating these alternative pathways and clarifying the broader molecular network involving EMP1-mediated regulation in liver injury. Furthermore, while this study establishes EMP1 as a novel marker and potential therapeutic target for HSC activation in MASLD-IRI, a direct comparative analysis of its sensitivity against classical HSC activation markers (α-SMA, COL1A2) is still lacking. Future clinical studies are warranted to evaluate their relative diagnostic or prognostic performance.

In conclusion, our findings highlight the therapeutic potential of targeting HSCs in MASLD liver transplantation. EMP1 expression may serve as a prognostic biomarker for post-transplant complications, and pharmacological inhibition of EMP1 or its interaction with TLN1 could attenuate HSC activation, thereby improving graft survival. These insights provide a mechanistic and translational framework for addressing the current challenges in using MASLD donor livers.

## Materials and methods

### Animal models

Multiple animal models were generated using a previously established approach [46, 48].

### MASLD rat model

Male rats were fed a high-fat methionine-choline deficient diet for 2 weeks from age 14 weeks onwards. Controls received normal chow. All animals were housed in pathogen-free conditions with ≤ 3 rats per cage.

### Liver fibrosis rat model

Male rats received injections of 2.5 mL/kg (20% in olive oil) twice weekly for 8 weeks from age 8 weeks onwards. Control mice received saline. Housing was pathogen-free with ≤ 3 rats per cage.

### Orthotopic liver transplantation rat model

Rats at 16 weeks of age were used. Buprenorphine was administered pre-surgery. Anesthesia was administered with pentobarbital sodium and the skin was cleaned with ethanol and betadine. The portal vein was skeletonized, livers perfused with UW solution, and excised. After 18 h of cold storage, livers were implanted. After 6 h reperfusion, blood was collected from the vena cava, livers were perfused with saline, excised, and mice were euthanized with isoflurane and cervical dislocation.

### Cell isolation, culture, and co-culture

Primary rat HSCs were isolated following the method established by Anabel Fernández-Iglesias et al. [61]. Primary rat HSCs were cultured in Dulbecco’s modified eagle medium (DMEM; Procell) supplemented with 10% (v/v) fetal bovine serum (FBS; Gibco, Waltham, MA, USA) and 100 units/mL penicillin/streptomycin (Gibco). All cell cultures were maintained at 37℃ with 5% carbon dioxide. Co-cultures of HSCs and LSECs were established according to Xiaoping et al. [62].

### Cell models

#### Hypoxia-reoxygenation cell model

HSC-T6, LX-2, rat LSECs, and rat kupffer cells were initially cultured in DMEM supplemented with 10% FBS and 4500 mg/L glucose to promote adherence. Hypoxia was induced by washing the cells with phosphate-buffered saline; the medium was replaced with glucose-free DMEM devoid of FBS and subsequently transferred to a hypoxic incubator (5% carbon dioxide, 94% N_2_, and 1% O_2_). After 10 h, the cells were washed again, and the medium was replaced with glucose-replete DMEM with 10% FBS for reoxygenation for 6 h. The cells were subsequently analyzed at designated time points.

#### MASLD cell model

HSC-T6, LX-2, rat LSECs, and rat kupffer cells were cultured for 48 h before being treated with a free fatty acid mixture (palmitic acid and oleic acid) at 1.5 mmol/L to induce steatosis over 48 h.

#### Human liver tissue and clinical data collection

Clinical data including age, sex, and medical history were documented for all participants upon admission. Blood samples were collected one day before surgery and on postoperative days 0, 1, 3, 5, and 7. During liver transplantation, portal vein blood and liver tissue samples were obtained 2 h after graft reperfusion. All donor livers were procured through the China Organ Transplant Response System between August 2022 and December 2024. Steatosis was defined as greater than 5% macrovesicular fat, as independently confirmed by two pathologists. The study included 18 patients who provided informed consent.

#### Cell transfection and injection of AAV

Lentivirus-shRNA, AAV6-shRNA, and plasmids were obtained from GeneChem. siRNA was obtained from GenePharma. Transfection was performed using Lipofectamine 2000 in Opti-MEM, which was replaced with DMEM containing 10% FBS after 6 h. Lentiviral transduction was performed with puromycin selection (5 μg/mL). For in vivo EMP1 knockdown, AAV6-shEMP1 (6 × 10^11^ vg) was injected via the tail-vein. Oligos are listed in Table S1.

#### Reagent treatment

TGF-β (HY-P70648), Defactinib (HY-12289A), CHX (HY-12320), and MG132 (HY-13259) were purchased from MedChemExpress. In cellular experiments, HSC activation was induced by serum-starvation overnight followed by treatment with 10 ng/mL TGF-β for 48 h. Defactinib (5 µM) was used as a FAK phosphorylation inhibitor by treating cells for 48 h. CHX and MG132 were used at a concentration of 20 µM, and the treatment time was determined according to the experimental design.

### Statistical analysis

Data are presented as mean ± SD. Analysis was performed using GraphPad Prism 8. Normality was assessed by Shapiro–Wilk test. For parametric data, analyses were performed using either an unpaired t-test (two groups), one-way ANOVA with Dunnett’s correction (multiple vs control), or two-way ANOVA with Bonferroni correction (multi-variable). For nonparametric data, analyses were performed using either the Mann–Whitney U test (two groups) or Kruskal–Wallis test with Dunnett’s correction (multiple groups). Significance was denoted as follows: *P < 0.05, **P < 0.01, ***P < 0.001; n.s., not significant.

## Supplementary Information

Supplementary Material 1.

## Data Availability

The datasets generated in the current study are available from the corresponding author upon reasonable request. In this study, the animal and cell RNA-seq data generated were deposited in the GEO (No. GSE254747) and the CNCB-NGDC (No.CRA030390) databases, respectively.
