# Supplementary material for: Epithelial membrane protein 1 drives hepatic stellate cell activation via the TLN1/FAK cascade in MASLD donor liver transplantation
Source: Mol Biomed. 2025 Nov 24;6:116. doi: 10.1186/s43556-025-00371-7 (PMC12644329; doi:10.1186/s43556-025-00371-7)
Supplement: Supplementary file 1 — Supplementary Material 1. [file 43556_2025_371_MOESM1_ESM.docx]

**Epithelial Membrane Protein 1 drives hepatic stellate cell activation via the TLN1/FAK cascade in MASLD donor liver transplantation**

Tongxi Li ^1*^, Ran Liu ^1*^, Huan Cao ^2*^, Shenghe Deng ^2^, Gengqiao Wang ^1^, Xueling Wang ^1^, Peng Zhao ^1^, Xuan Li ^1^, Jingjin Zhu ^1^, Shuyu Shao ^1^, Hao Chen ^1^, Lei Liu ^1^, Chen Zhang ^2^, Chuanzheng Yin^1#^, Zifang Song ^1#^

^1^ Department of Hepatobiliary Surgery, Union Hospital, Tongji Medical College, Huazhong University of Science and Technology, Wuhan, Hubei, China.

^2^ Center for Liver Transplantation, Union Hospital, Tongji Medical College, Huazhong University of Science and Technology, Wuhan, Hubei, China.

*Tongxi Li, Ran Liu and Huan Cao contributed equally to this work.

**Supplementary directory**

Figures and Legends ............................................................................................ **2**

Methods ............................................................................................................... **13**

Tables ................................................................................................................... **17**

**Supplementary Figures and Legends**

**
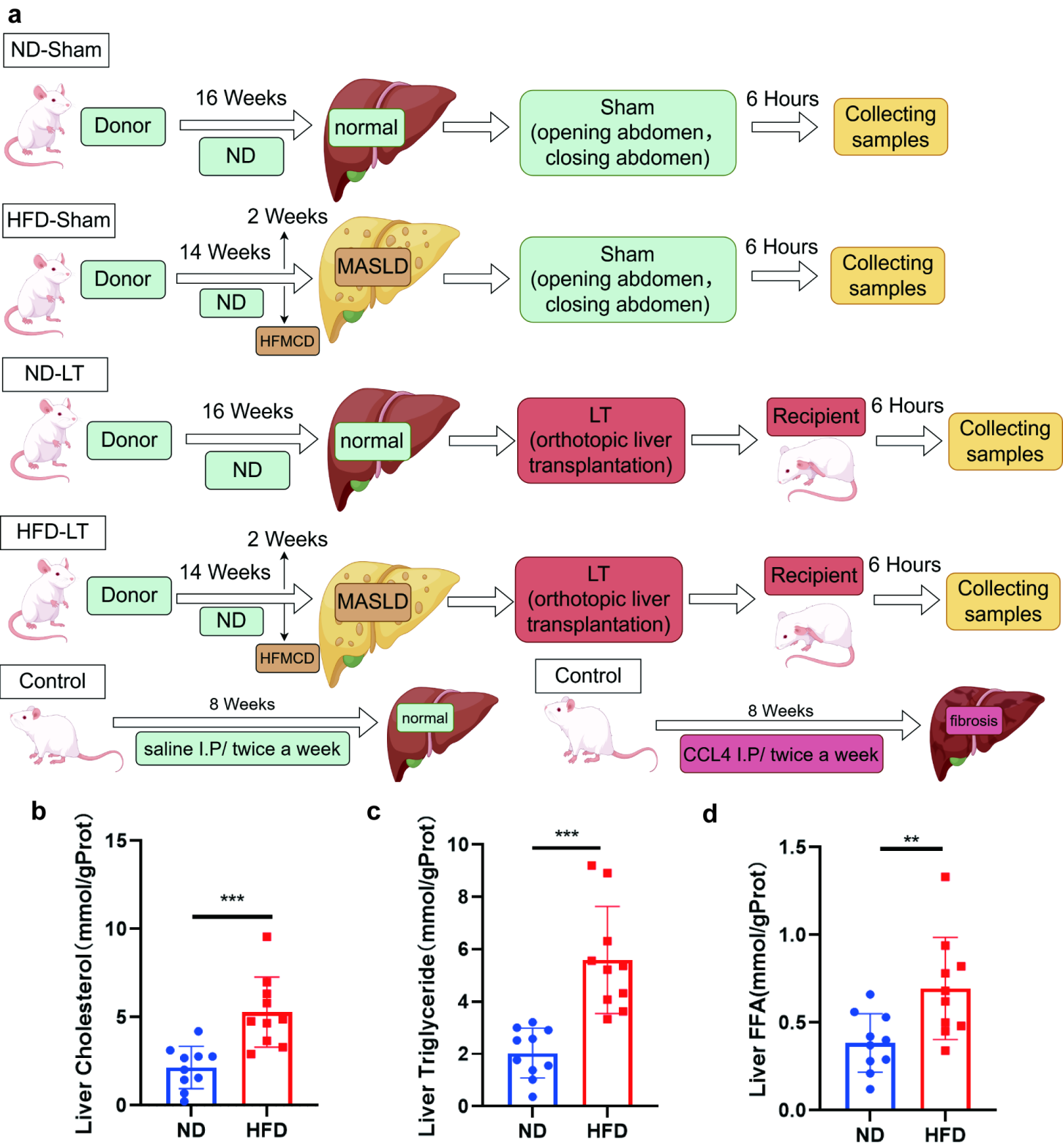
**

**Fig.S1 Animal model construction and validation.**

**a.**Flowchart of animal model construction. **b-d.** Detection of liver tissue TC, TG, and FFA to confirm the establishment of MASLD model (n=10/group).


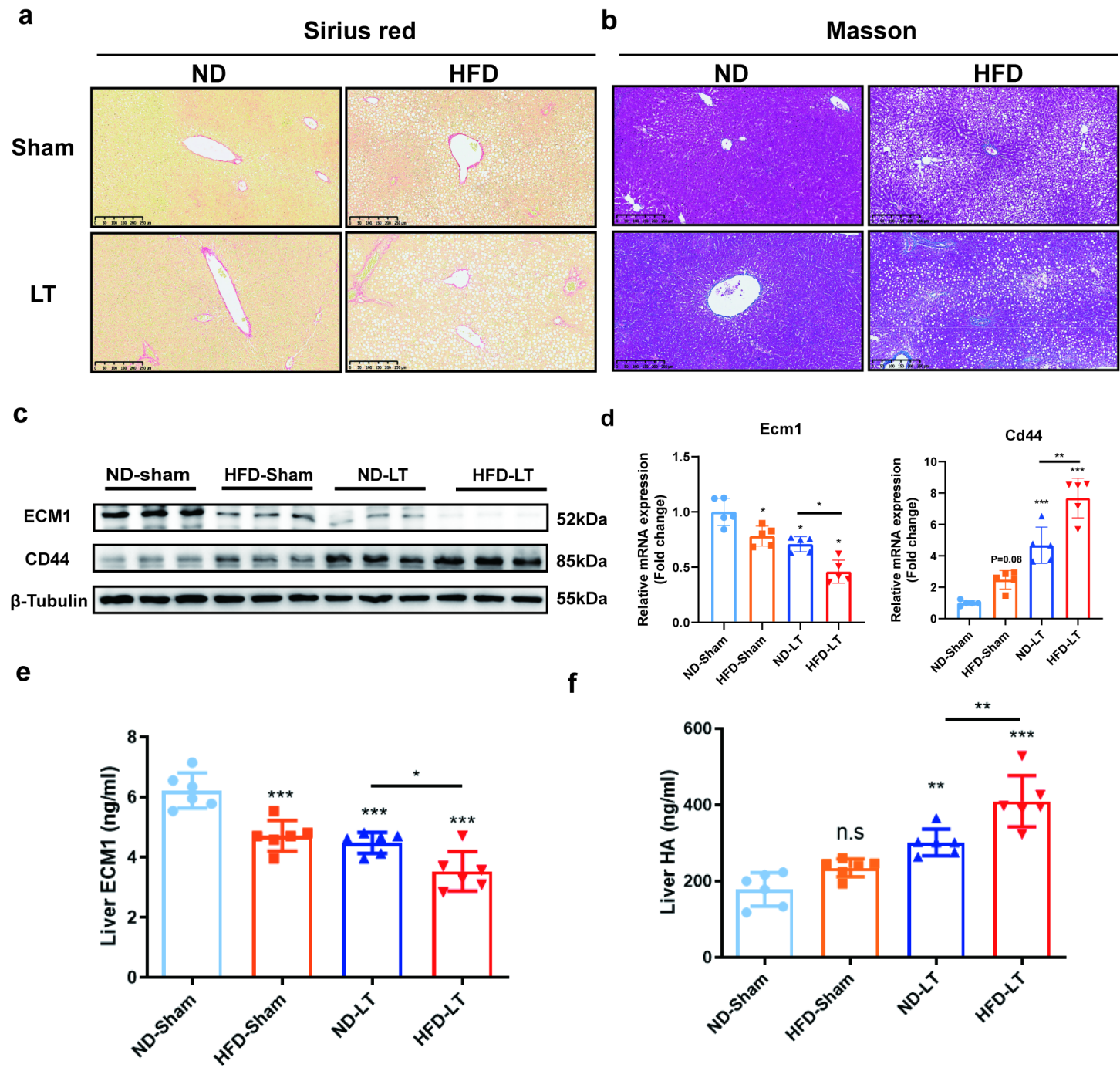


**Fig.S2 MASLD-IRI leads to fibrosis susceptibility but does not rapidly lead to liver fibrosis.**

**a,b.** Pathological detection of hepatic fibrosis in rat liver tissues stained with Sirius red and Masson staining showing the degree of fibrosis in the liver (n=3/group). The scale bar =250 μm. **c,d.** Expression of ECM1, CD44 protein(n=3/group) and mRNA (n=5/group) were detected in liver. **e,f.** ECM1 and HA synthesis were detected in rat liver tissues using ELISA (n=5/group). /group). All data used for statistics above are SD ± mean, n.s > 0.05, * P < 0.05, ** P < 0.01, *** P < 0.001, analyzed by two-way ANOVA or t-test followed by Tukey test.


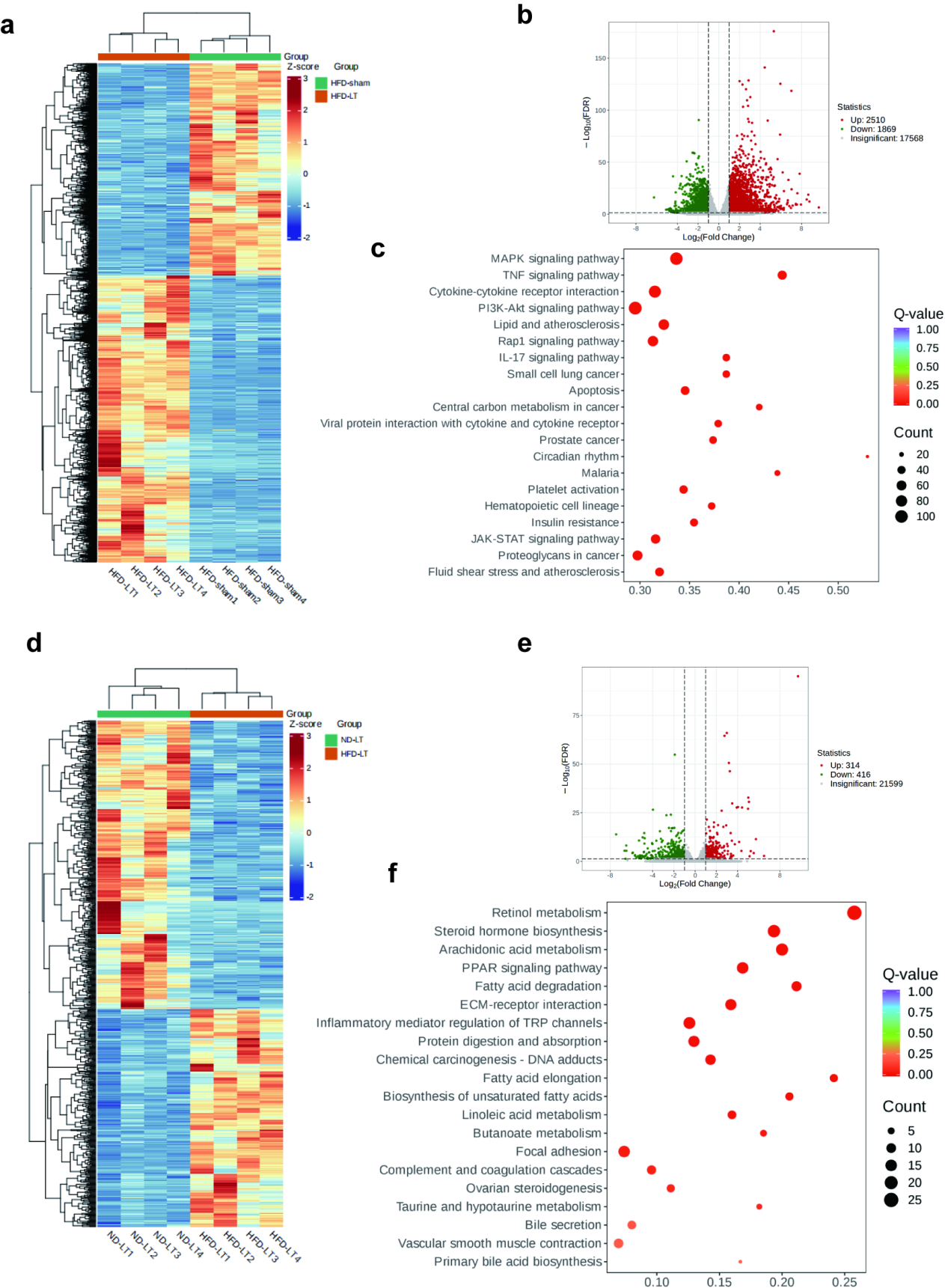


**Fig. S3 transcriptome sequencing and enrichment in different groups.**

**a-f.** Comparison of transcriptome sequencing differences and KEGG enrichment analysis between subgroups (n=4/group) .


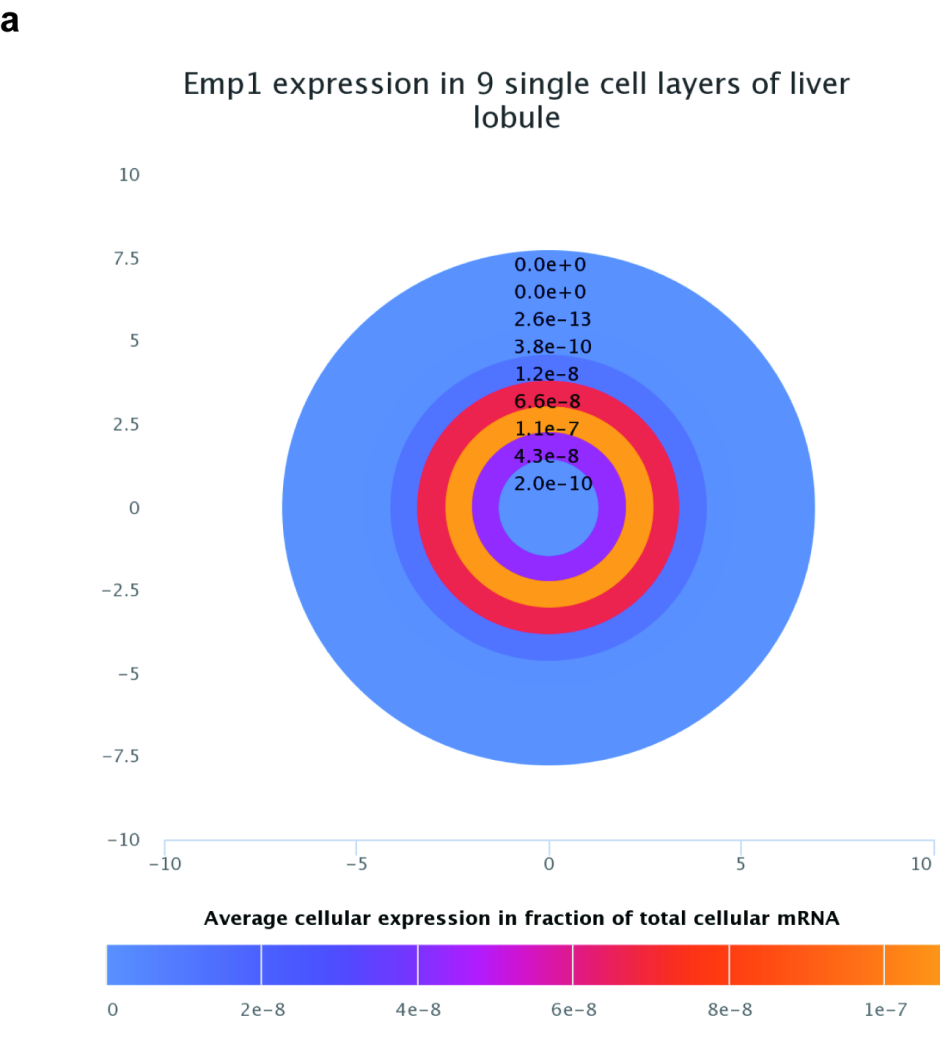


**Fig. S4 Localization of EMP1 in liver lobular compartmentation.**

**a.** Localization of EMP1 in the liver was analyzed by raw letter based on single-cell-space transcriptomics and hepatic lobular stratification theory.


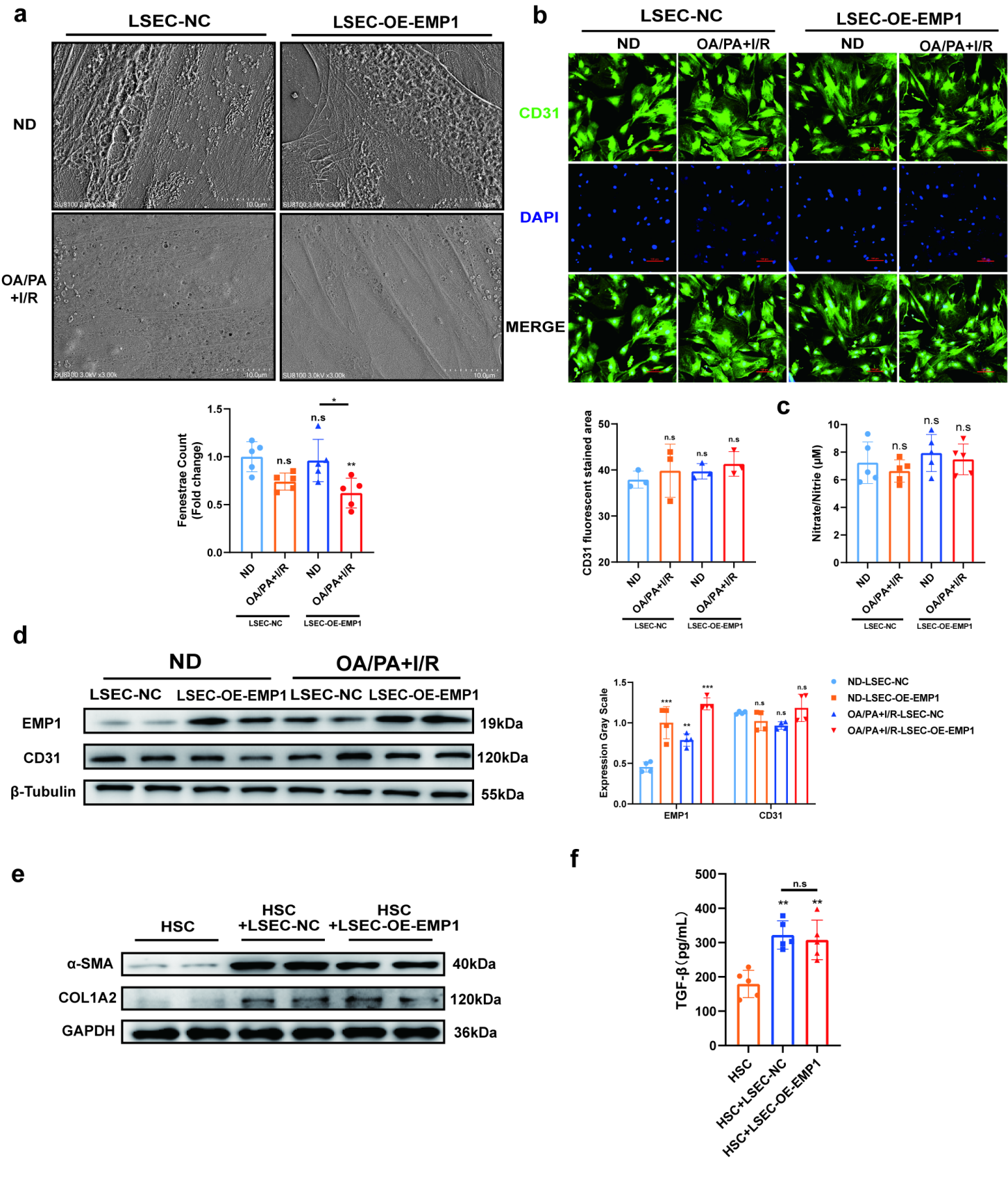


**Fig. S5 EMP1 overexpression in LSECs modulates their autocrine signaling and paracrine activation of HSCs.**

**a.** Transmission electron microscopy photographs of LSECs under the regulation of EMP1 expression given to ND and OA/PA+I/R groups (n=3/group)**. b.** Fluorescence staining of CD31, a marker of capillarization in LSECs (n=3/group)**; c.** Biochemical assay for Nitrate/Nitrite in different groups (n=5/group)**. d.** Protein assay for EMP1 in LSECs in each group and capillarization marker CD31 (n=4/group)**. e.** Co-culture of HSCs with different groups of LSECs for protein assay of HSC activation markers (n=4/group)**. f.** ELISA for secreted TGF-β in the co-culture system (n=5/group)**.** All the above data used for statistics are SD ± mean, n.s > 0.05, * P < 0.05, ** P < 0.01, *** P < 0.001, and were analyzed by two-way ANOVA or t-test followed by Tukey test.


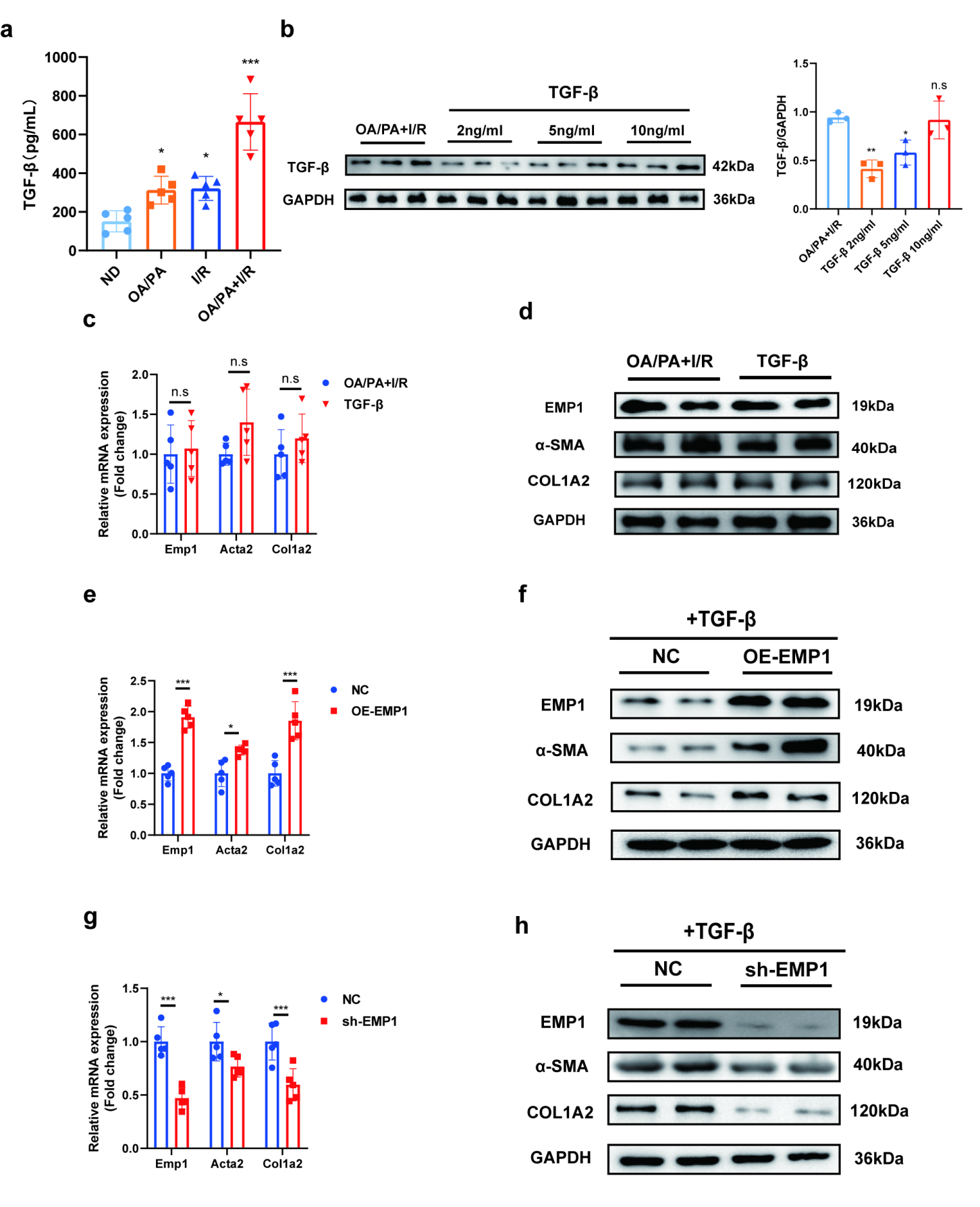


**Fig. S6 Rationalization of cell model construction and validation of transfection.**

**a.**The secretion of TGF-β induced by different models at cellular level was detected by ELISA(n=5/group). **b.** Detection of TGF-β levels in OA/PA+IRI models with different concentrations of TGF-β addition by protein expression(n=3/group). **c,d.** Detection of differences in the expression levels of EMP1, α-SMA, and COL1A2 by the two models inducing HSC activation(n=4/group). **e,f.** Verification of the transfection transfection potency of OE-EMP1 and the ability to regulate HSC activation by detecting the mRNA (n=5/group) and protein expression levels of EMP1, α-SMA, and COL1A2(n=4/group). **g,h.** Verification of the transfection transfection potency and the ability to regulate HSC activation by detecting the expression levels of EMP1, α-SMA, COL1A2. mRNA (n=5/group) and protein expression levels(n=4/group) to verify the transfection transfection potency of sh-EMP1 and the ability to regulate HSC activation. All the above data used for statistics are SD ± mean, n.s > 0.05, * P < 0.05, ** P < 0.01, *** P < 0.001, and were analyzed by two-way ANOVA or t-test followed by Tukey test.


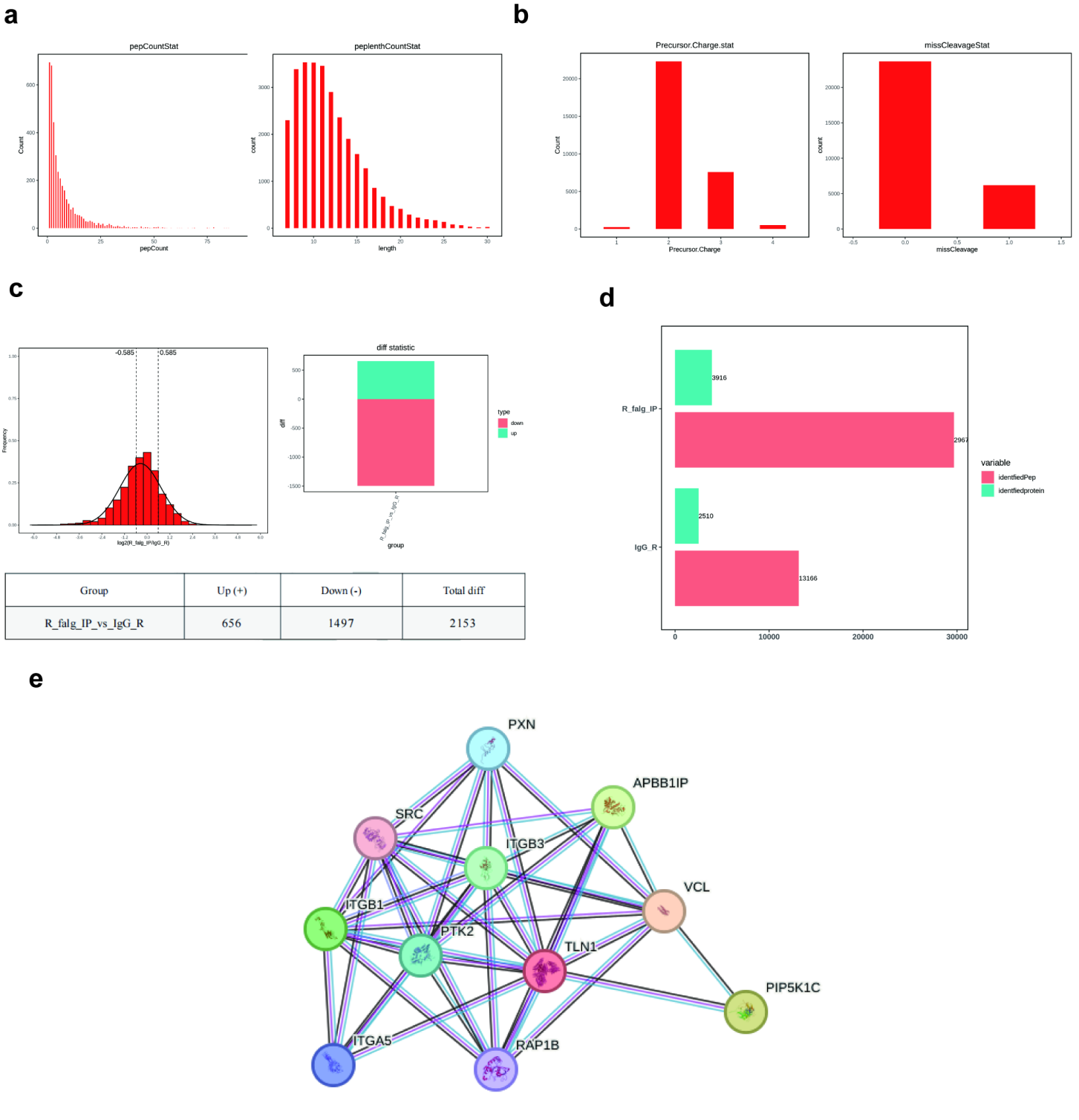


**Fig.S7 Information processing and discrepancy detection in CoIP/MS joint STRING.**

**a.** Amino acid length and peptide number distribution of identified peptides. **b.** Distribution of missed cleavage sites. **c.** Quantitative ratio distribution graph and differential protein screening and statistics. **d.** Protein quantitative quantity statistics graph. **e.** STRING protein interaction network diagram showing the interaction of TLN1 with PTK2 (FAK).


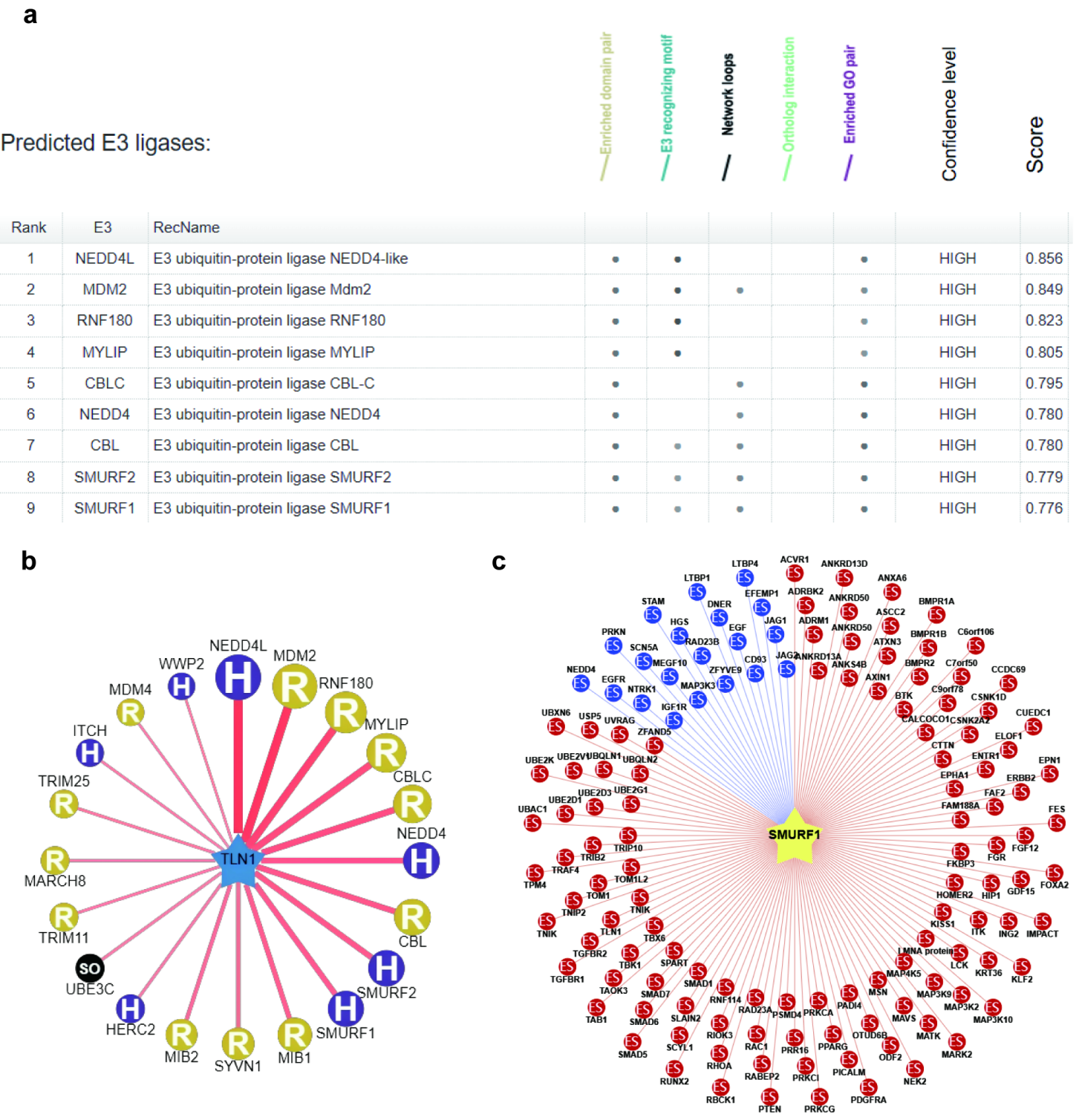


**Fig.S8 Binding protein prediction of E3 ubiquitin ligases that may bind to TLN1 with SMURF1.**

**a,b.** Analysis of E3 ubiquitin ligases that may bind to TLN1 using UbiBrowser 2.0. **c.** SMURF1 as a common E3 ubiquitin ligase was predicted to be ubiquitinated with a wide range of SMAD family, TGF-β pathway, MAPK pathway, and RAC pathway proteins. pathway proteins to undergo ubiquitination.


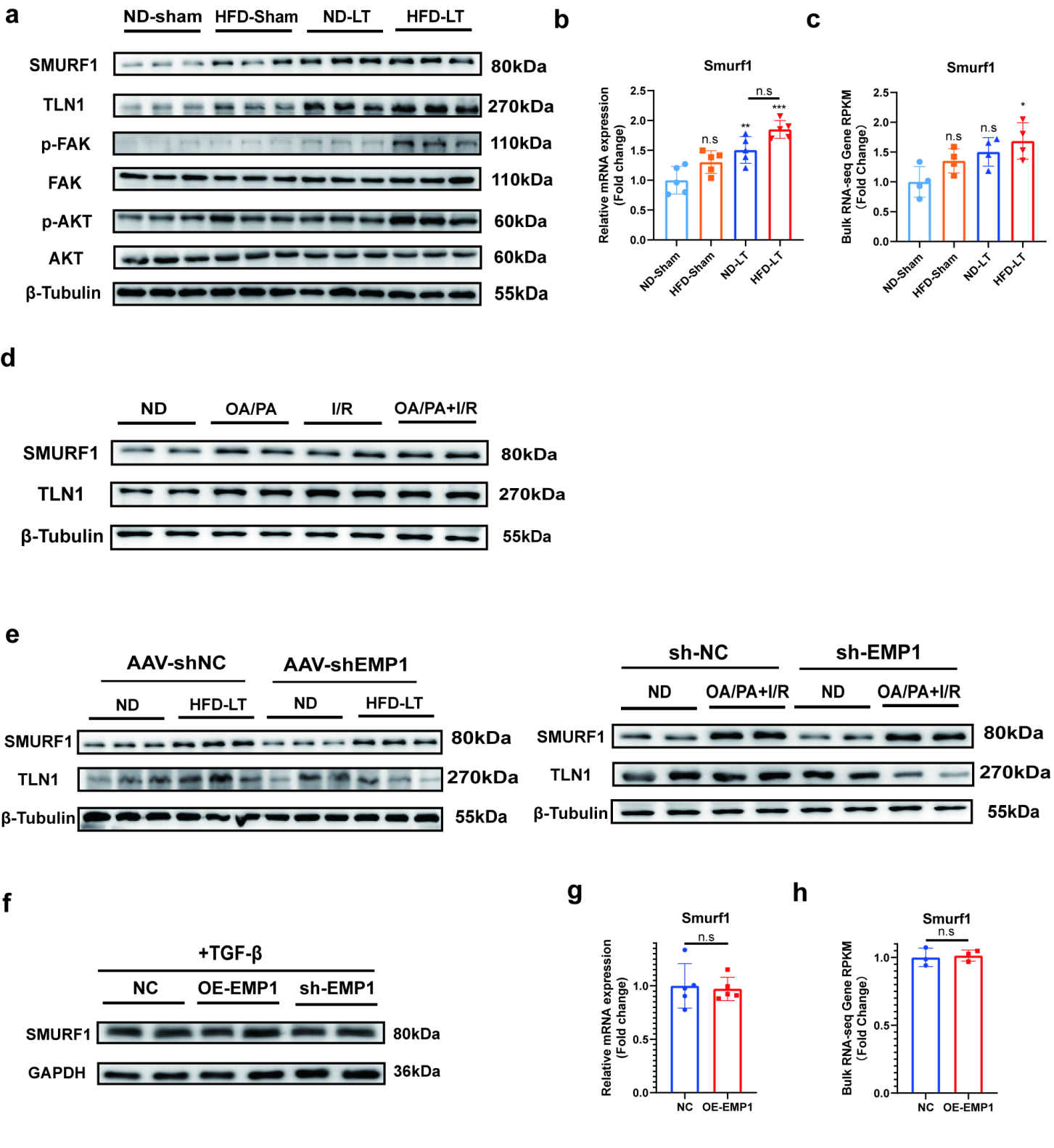


**Fig.S9 MASLD-IRI upregulates SMURF1, but its ubiquitination degradation of TLN1 is inhibited by EMP1.**

**a.** Changes in protein expression and phosphorylation levels of SMURF1, TLN1/FAK/AKT axis were verified in animal models(n=3/group) . **b,c.** mRNA expression of SMURF1 was verified in animal models using qRT-PCR (n=5/group) and transcriptome sequencing with RPKM values (n=4/group). **d.** HSC-T6 was simulated using OA/PA+I/R, respectively. MASLD and IRI to detect changes in SMURF1 and TLN1 protein expression (n=4/group) . **e.** TLN1 and SMURF1 protein expression assays in MASLD-IRI animal (n=3/group) and cell (n=4/group) models with and without EMP1 silencing. **f-h.** Changes in protein expression of SMURF1 under the regulation of EMP1 by using WB (n=4/group), qRT-PCR (n=5/group), and transcriptome sequencing RPKM values (n=3/group). All the above data used for statistics are SD ± mean, n.s > 0.05, * P < 0.05, ** P < 0.01, *** P < 0.001, analyzed by two-way ANOVA or t-test followed by Tukey test.


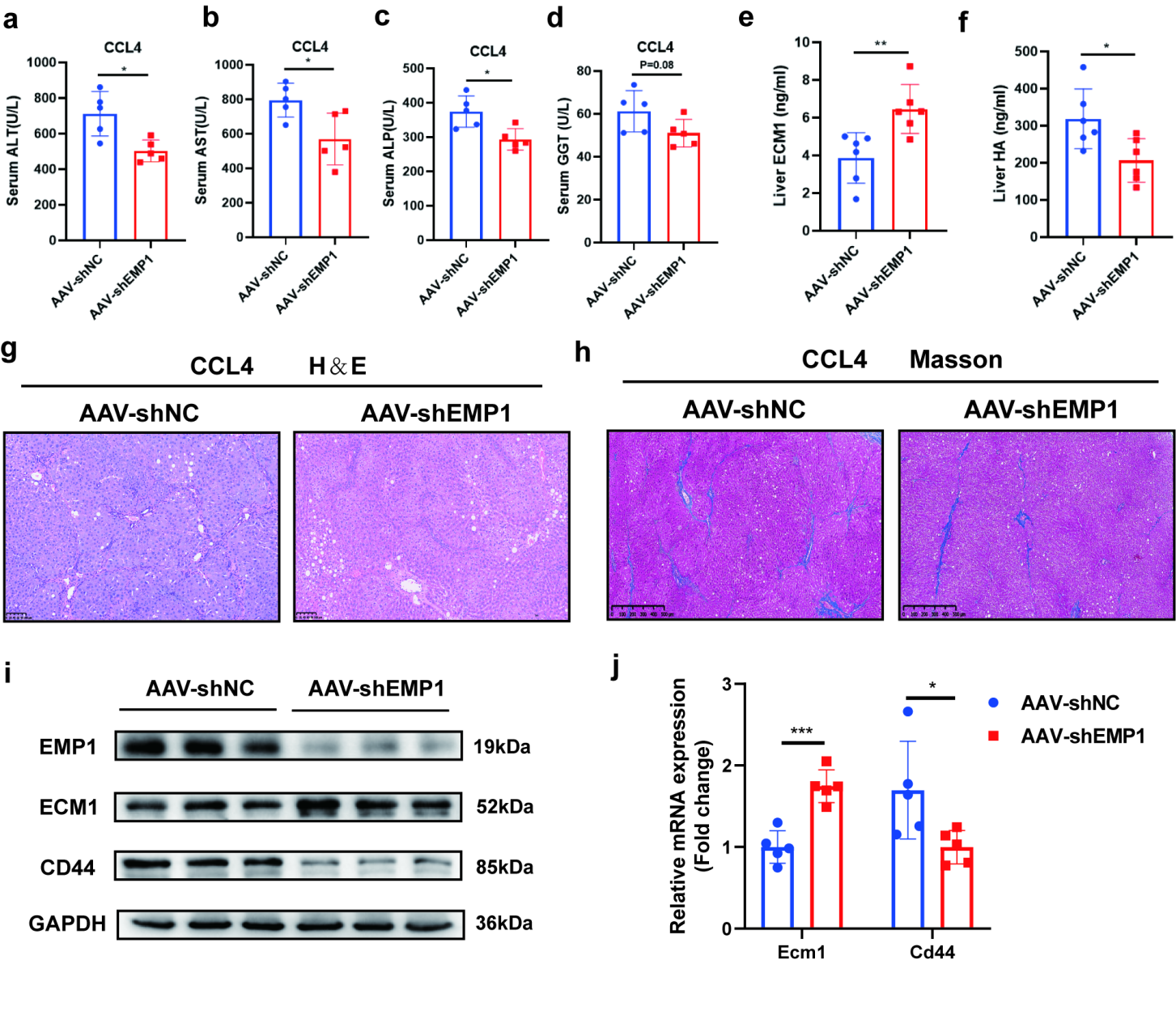


**Fig. S10 EMP1 mediates HSC activation modulation also occurs in liver fibrosis.**

Liver fibrosis model was constructed by intraperitoneal injection with CCL4.

**a-d.** Serum liver injury markers ALT, AST, ALP, and GGT were detected to check the overall degree of liver injury (n=5/group). **e,f.** The amount of ECM1 and HA synthesized in rat liver tissues was detected by using ELISA (n=5/group). **g,h.** Pathological examination of rat liver tissues was performed to observe the type of liver injury, and H＆E shows tissue edema, structural disorders, and necrosis of the liver (n=3/group),The scale bar =100 μm.Masson staining demonstrates collagen fiber formation, i.e., the degree of hepatic fibrosis (n=3/group),The Scale bar = 500 μM, shown in the lower left corner. **i,j.** ECM1 and HA synthesized in liver of rats (n=5/group). “protein ECM1 and ECM synthesis-associated CD44 protein(n=3/group) and mRNA (n=5/group) expression assays. All data used for statistics above are SD ± mean, n.s > 0.05, * P < 0.05, ** P < 0.01, *** P < 0.001, and were analyzed by two-way ANOVA or t-test followed by Tukey test.

**Supplementary Methods**

**Immunoblotting and Immunofluorescence.**

Tissues or cells are lysed on ice using radioimmunoprecipitation assay lysis buffer containing 1% protease and phosphatase inhibitors. Cell lysis products were centrifuged (4°C, 12,000 rpm, 15 min) and the supernatant collected. Protein content was determined by dicinchoninic acid assay. The obtained proteins were added to 5× loading buffer and heated at 95°C for 10 min and used for Western blot analysis.Multiplex immunofluorescence was performed as follows: dewaxed antigenic repair of Liver sections and endogenous enzyme blocking with H2O2 was performed for 30 min then blocked with 10% goat serum (37°C, 30 min). Primary antibodies were diluted according to the experimental concentRation and incubated overnight at 4°C. Horseradish peroxidase (HRP) enzyme-labeled secondary antibodies were prepared using PBS with Tween 20 (PBST) (37°C, 1 h). Tyramine signal amplification (TSA) reagent was added to the tissue (37°C, 1 h) for antigen repair and serum blockade. Then, target antibody, HRP enzyme-labeled secondary antibody and TSA reagent were added successively for antigen repair and serum blocking. After the addition of target antibody, DAPI was added for nuclear staining. Detailed antibody information used in this experiment is provided in Supplementary file 1: Table 2.

**Co-immunoprecipitation and ubiquitination assays.**

For the immunoprecipitation (CoIP) assay, the protein supernatant was collected in the same manner as described above. After adding the target antibody and protein A + G agarose (Beyotime, P2012) to the collected protein supernatant, the agarose beads were cleaned overnight at 4°C, then added to the uploading buffer and heated at 95°C for 10 min. As for the ubiquitination assays, different plasmids were transfected according to the requirements of different experiments and treated with TGF-β and MG132. Cells were collected and prepared into protein samples according to the above CoIP method, sepaRated by appropriate SDS/PAGE gels, and finally assayed.

**RNA extraction and quantitative real-time polymerase chain reaction (qRT-PCR).**

Total RNA was extracted from tissues and cells using TRIzol reagent (Invitrogen, USA) and the RNApure TissueCell Kit (CWBIO, CHN), and RNA concentRation was determined using a spectrophotometer. RNA samples (1 μg) were reverse transcribed to cDNA using the Reverse Transcription Kit (Vazyme, CHN). qRT-PCR was performed using the Super SYBR Green Kit (Vazyme, CHN, USA). Primer sequences for these genes are listed in Supplementary file 1: Table 3

**Enzyme-linked immunosorbent assay(ELISA).**

TGF-β(ml107101)、ECM1(ml098167), HA(ml985677) ELISA kits were purchased from Mlbio (Shanghai, China) and used according to the instructions of the kit manufacturers. Absorbance (OD) values at different wavelengths of excitation light were measured using an enzyme marker. The standard curve was plotted according to the kit protein standards, and the expression of target proteins was calculated using ELISACalc and the kit instructions, and finally statistically analyzed by Graphpad Prism.

**Staining and analysis.**

**H＆E、Sirius Red and Masson staining.**

H&E staining was performed by dewaxing with xylene and dehydRating with ethanol followed by hematoxylin staining for 20 min at room tempeRature, eosin staining for 2 min, followed by ethanol dehydRation, and finally sealing with neutral gel. Each process was strictly washed with distilled water.Sirius Red and Masson staining was accomplished in similar steps depending on the staining solution required.

**TUNEL staining.**

For TUNEL stain The liver tissues collected were fixed in 4% paraformaldehyde and subsequently underwent processing for paraffin embedding. The detection of dead cells with DNA fragmentation was performed through the TUNEL assay, utilizing the In Situ Cell Death Detection Kit, POD (Roche), following the manufacturer's protocol. TUNEL positivity was analyzed with Fiji software.

**Biochemical Analysis.**

Serum levels of alanine aminotransferase (ALT) and aspartate aminotransferase (AST) were determined using a Cobas C111 biochemical analyzer (Roche), following the manufacturer's instructions. Serum or liver ALP (A059-2-2), GGT (C017-2-1), SOD (A001-3-2), MDA (A003-1-2), TC (A111-1-1), TG (A110-1-1), FFA (A042-2-1) , were determined by purchasing Jiancheng The assays were performed by purchasing commercialized kits produced by Bioengineering Institute (Nanjing, China). Nitrate/Nitrite kits (S0023) produced by Beyotime. The assay procedure was performed according to the commercial instruction manual, and the expression of target proteins was calculated by measuring the absorbance (OD) value of the enzyme marker and the standard curve determined by the standard samples of the kits, and finally the statistical analysis was completed by Graphpad Prism.

**Transcriptome profiling.**

RNA is extracted from tissues or cells, and the RNA samples are subsequently subjected to stringent quality control, mainly by means of the Agilent 2100 bioanalyzer: to accuRately detect RNA integrity. The first is to enrich mRNA with polyA tails by Oligo(dT) magnetic beads, taking advantage of the structural feature that most of the mRNAs in eukaryotes have polyA tails. mRNA was enriched from total RNA by using Oligo dT beads. mRNAs were enriched from total RNA by using Oligo dT beads, and after fragmentation, the first-strand cDNAs were synthesized by using random hexameric primers, and the second-strand cDNAs were subsequently synthesized. After end repair, A-tail addition, junction ligation, fragment selection, amplification and purification, the library was ready. After the library construction was completed, preliminary quantification was performed using Qubit2.0 Fluorometer, and the library was diluted to 1.5 ng/ul. Subsequently, the insert size of the library was detected using Agilent 2100 bioanalyzer, and after the insert size was as expected, the effective concentRation of library was accuRately quantified by qRT-PCR (the effective concentRation of library was higher than that of the library). quantification (the effective concentRation of the library was higher than 1.5 nM) to ensure the quality of the library. The library was pooled according to the effective concentRation and the target downstream data volume for Illumina sequencing. Four types of fluorescently labeled dNTP, DNA polymerase, and junction primers are added to the sequencing flow cell for amplification. When extending the complementary strand of each sequencing cluster, each addition of fluorescently labeled dNTP releases corresponding fluorescence, and the sequencer captures the fluorescence signals and converts the light signals into sequencing peaks through the computer software, thus obtaining the sequence information of the fragment to be tested. Data were collected by Illumina X-ten platform software HCS version 3.3.76 (Illumina). Transcriptome mapping analysis was assisted by Metware.

**CoIP/MS.**

The area where the protein is located in the SDS-PAGE was scooped out and cut into small pieces of about 1 cm^2^; add double-distilled water, 50% ACN/100 mM NH4HCO3 (pH 8.0) to wash sequentially and then vacuum-dried. Add 10 mM DTT/50 mM NH4HCO3 (pH 8.0) solution to the pellet, and incubate at 56℃ for 1h for reduction reaction. Subsequently, 55 mM iodoacetamide/50 mM NH4HCO3 (pH 8.0) solution was added, and the reaction was placed at room temperature and in the dark for 30 min, and washed by adding ACN again and then vacuumed dry. Add trypsin, 50 mM NH4HCO3 solution and incubate at 37℃ overnight for digestion. Then, 60% ACN/5% formic acid was added as extraction solution and ultrasonicated for 10 min. After centrifugation, the supernatant was aspirated, and the peptide was desalted using a C18 column to complete the sample preparation. The mass spectrometry data were collected using a Q Exactive HF mass spectrometer coupled with an UltiMate 3000 RSLCnano liquid phase liquid chromatography system. Inhalation by the autosampler was followed by separation on an analytical column. An analytical gradient was established using two mobile phases. The flow rate of the liquid phase was set to 300 nL/min. The mass spectra were acquired in DDA mode, with each scan cycle consisting of a full MS scan (R = 60 K, AGC = 3e6, max IT = 25 ms, scanrange = 350-1500 m/z) and 20 subsequent MS/MS scans (R = 15 K, AGC = 1e5, max IT = 1e5, max IT = 25 ms). AGC = 1e5, maxIT = 50 ms). The mass spectrometry data were searched by MaxQuant (V1.6.6) software using the database search algorithm Andromeda.The databases used for the searches were: custom proteome reference database 23103020 (with 1 protein sequence), and the proteome reference database of Rattus_norvegicus in Uniprot. The search results were filtered by 1% FDR at the protein and peptide level, and the entries of antic library proteins, contaminated proteins, and proteins with only one modified peptide were deleted, and the remaining identification information was used for subsequent analysis.

**Bioinformatic Analysis.**

**GEPIA2.**

The expression correlations among multiple proteins of interest in human liver tissues were assessed using the Gene Expression Profiling Interactive Analysis 2 (GEPIA2) platform (http://gepia2.cancer-pku.cn). This web server provides extensive RNA-seq data derived from large-scale genomic databases, including both normal and disease tissue samples. We selected “Liver” as the tissue of interest and input the official gene symbols of the target proteins. Spearman’s rank correlation was employed to evaluate co-expression relationships. Based on the calculated correlation coefficient (R-value), we defined strong positive correlation as R > 0.8, moderate positive correlation as R between 0.5 and 0.8, and weak or negligible correlation as R < 0.3. Statistical significance was set at p < 0.05. The results were presented as scatterplots generated by GEPIA2, which visually and quantitatively illustrate the pairwise associations between transcripts. This analysis provided insight into potential regulatory interactions and functional linkages among key proteins involved in hepatic physiology and pathology at the transcriptional level.

****CellBrowser.****

Single-cell transcriptomic profiling of liver tissues was conducted using the UCSC CellBrowser ([https://cells.ucsc.edu](https://cells.ucsc.edu/)). Publicly available human liver scRNA-seq datasets were accessed through the platform. Cell type annotation was performed based on established marker genes. Dimensionality reduction and visualization were carried out using UMAP. Gene expression patterns across different cell types were explored using built-in visualization tools such as heatmaps and feature plots. This analysis enabled the identification of cell-type-specific expression profiles of target genes within the hepatic microenvironment, providing insight into their potential roles across distinct cellular populations.
**UbiBrowser 2.0.**

To predict potential E3 ubiquitin ligases and ubiquitination-related protein interactors of TLN1, we performed a bioinformatic analysis using UbiBrowser 2.0 (http://ubibrowser.bio-it.cn/ubibrowser_v2/), a public platform dedicated to predicting ubiquitination regulatory networks.The system was queried with default parameters, which integrate multiple predictive algorithms including domain-domain interaction, motif recognition, and co-expression evidence. Results were filtered with a confidence score threshold of ≥ 0.7 to ensure high-reliability predictions. Result visualization and functional annotation were further supported by built-in tools within UbiBrowser 2.0.
**STRING.**

To systematically predict and visualize functional interactions associated with TLN1, we performed a protein-protein interaction (PPI) network analysis using the STRING database (version 12.0, https://string-db.org). The search was configured to include only interactions with a minimum confidence score of 0.7 (high confidence). Active interaction sources included experiments, databases, co-expression, and text mining, while curated pathways and functional annotations were incorporated for downstream enrichment analysis. The resulting network was visualized within the STRING environment, with nodes representing proteins and edges indicating functional or physical associations.

**Supplementary Tables**

**Supplemental Table 1. Basic characteristics of the donors.**

| Characteristics | Steatotic liver | | P value |
| --- | --- | --- | --- |
|  | No (n=10) | Yes (n=8) |  |
| **Gender** |  |  | 0.502 |
| Male | 6 (60.0%) | 6 (75%) |  |
| Female | 4 (40.0%) | 2 (25%) |  |
| **Age, *y*** | 50.7 (42.0-63.0) | 48.1 (39.0-53.0) | 0.478 |
| **Height, *cm*** | 167.3 (155.0-175.0) | 167.6 (160.0-179.0) | 0.931 |
| **Weight, *kg*** | 65.0 (32.0-92.0) | 73.4 (57.2-94.5) | 0.119 |
| **Macrosteatosis** | 0 | 50% (5%-40.0%) | <0.001 |
| **Pretransplant laboratory values** |  |  |  |
| White blood cell | 10.7 (3.5-17.7) | 12.2 (7.7-14.9) | 0.427 |
| Red blood cell | 3.8 (2.7-5.2) | 3.9 (2.7-5.3) | 0.826 |
| Hemoglobin | 124.0 (80.0-172.0) | 112.0 (87.0-139.0) | 0.334 |
| Platelets | 195.6 (58.0-328.0) | 149.2 (56.0-226.0) | 0.272 |
| Lymphocyte | 0.8 (0.5-1.3) | 1.1 (0.2-2.0) | 0.840 |
| Mononuclear cell | 0.8 (0.3-1.4) | 0.8 (0.3-1.5) | 0.778 |
| Neutrophil | 8.8 (2.5-16.6) | 10.2 (6.2-12.5) | 0.374 |
| Eosinophilic cell | 0.1 (0.0-0.4) | 0.1 (0.0-0.2) | 0.787 |
| Basophil cell | 0.0 (0.0-0.1) | 0.0 (0.0-0.2) | 0.934 |
| Lymphocyte% | 9.3 (2.8-15.8) | 8.5 (1.9-16.6) | 0.681 |
| Monocyte% | 7.6 (3.8-12.7) | 7.0 (2.9-12.0) | 0.642 |
| Neutrophil% | 81.9 (71.2-87.7) | 83.7 (81.5-95.2) | 0.556 |
| Eosinophil% | 1.1 (0.0-3.2) | 0.5 (0.0-1.0) | 0.316 |
| Basophil% | 0.2 (0.0-0.5) | 0.2 (0.0-1.0) | 0.719 |
| C-reactive protein | 102.8 (2.3-402.6) | 362.8 (3.6-770.9) | 0.130 |
| PCT | 3.4 (0.1-13.2) | 20.5 (0.2-100.3) | 0.129 |
| alkaline phosphatase | 79.4 (49.0-162.0) | 80.3 (40.0-126.0) | 0.968 |
| Total protein | 65.6 (37.8-83.0) | 60.3 (27.6-90.8) | 0.731 |
| Albumen | 39.9 (17.4-64.7) | 36.2 (16.3-53.5) | 0.613 |
| Globulin | 29.4 (18.3-39.6) | 30.9 (24.0-54.3) | 0.899 |
| Albumin/Globulin | 1.4 (0.8-2.6) | 1.3 (0.6-1.8) | 0.796 |
| γ-glutamyl transferase | 60.6 (1.0-181.0) | 70.2 (14.0-160.0) | 0.843 |
| AST | 58.4(12.0-182.0) | 97.6 (30.2-229.8) | 0.132 |
| ALT | 57.7 (4.0-253.2) | 126.4 (15.0-313.0) | 0.118 |
| Total bilirubin | 14.7 (5.7-24.7) | 18.4 (5.0-32.3) | 0.272 |
| Direct bilirubin | 8.4 (2.6-18.2) | 11.7 (3.7-22.9) | 0.222 |
| Total bile acid | 6.0 (2.0-12.0) | 7.2 (2.2-12.2) | 0.617 |
| Potassium | 3.9 (1.6-5.0) | 4.6 (2.6-6.1) | 0.210 |
| Urea nitrogen | 9.9 (3.0-26.9) | 14.9 (3.1-28.2) | 0.374 |
| Creatinine | 107.0 (45.0-197.0) | 146.4 (60.2-307.0) | 0.407 |
| Uric acid | 231.2 (85.3-363.0) | 253.3 (76.3-417.7) | 0.466 |
| PT | 12.6 (9.6-15.7) | 13.9 (9.3-18.8) | 0.352 |
| APTT | 35.2 (11.5-65.6) | 37.6 (13.7-60.1) | 0.735 |
| TT | 17.0 (6.5-17.2) | 17.5 (10.7-20.8) | 0.946 |
| INR | 1.2 (0.8-2.2) | 1.3 (0.9-1.8) | 0.528 |

**Supplemental Table 2. Basic characteristics of the recipients.**

| Characteristics | Steatotic liver | | P value |
| --- | --- | --- | --- |
|  | No (n=10) | Yes (n=8) |  |
| **Gender** |  |  | 0.740 |
| Male | 7 (70.0%) | 5 (62.5%) |  |
| Female | 3 (30.0%) | 3 (37.5%) |  |
| **Age, *y*** | 49.9 (27.0-70.0) | 47.8 (31.0-60.0) | 0.737 |
| **Height, *cm*** | 168.2 (159.0-178.5) | 165.8 (158.0-175.0) | 0.518 |
| **Weight, *kg*** | 66.2 (49.2-85.8) | 63.1 (48.0-81.4) | 0.377 |
| **Pretransplant laboratory values** |  |  |  |
| White blood cell | 8.5 (2.0-19.1) | 6.8 (3.5-10.2) | 0.439 |
| Red blood cell | 3.8 (2.3-4.3) | 3.9 (2.7-5.5) | 0.548 |
| Hemoglobin | 116.4 (77.0-157.0) | 113.1 (89.0-132.0) | 0.780 |
| Platelets | 147.1 (38.0-401.0) | 131.1 (52.0-295.0) | 0.744 |
| Lymphocyte | 0.4 (0.1-1.0) | 0.5 (0.3-1.3) | 0.963 |
| Mononuclear cell | 0.6 (0.1-1.8) | 0.8 (0.2-2.2) | 0.592 |
| Neutrophil | 8.6 (1.1-17.1) | 10.4 (3.3-22.8) | 0.516 |
| Eosinophilic cell | 0.1 (0.0-0.4) | 0.1 (0.0-0.3) | 0.805 |
| Basophil cell | 0.0 (0.0-0.1) | 0.1 (0.0-0.3) | 0.675 |
| Lymphocyte% | 7.0 (2.0-18.9) | 7.6 (2.9-15.1) | 0.806 |
| Monocyte% | 7.0 (1.3-14.9) | 7.1 (3.5-13.8) | 0.941 |
| Neutrophil% | 87.3 (73.0-95.2) | 87.0 (79.6-92.3) | 0.934 |
| Eosinophil% | 1.0 (0.0-2.0) | 0.6 (0.0-1.3) | 0.304 |
| Basophil% | 0.2 (0.0-0.7) | 0.2 (0.0-0.9) | 0.765 |
| C-reactive protein | 451.6 (3.2-2012.5) | 652.7 (4.2-2522.5) | 0.614 |
| PCT | 9.4 (0.1-18.5) | 13.5 (0.2-25.3) | 0.117 |
| alkaline phosphatase | 166.8 (39.0-405.0) | 147.3 (28.0-335.0) | 0.735 |
| Total protein | 59.8 (45.1-81.6) | 56.2 (30.6-79.5) | 0.731 |
| Albumen | 30.9 (17.4-38.8) | 33.4 (14.7-47.2) | 0.526 |
| Globulin | 23.8 (12.8-45.1) | 22.8 (14.2-35.6) | 0.836 |
| Albumin/Globulin | 1.3 (0.5-2.7) | 1.5 (1.0-2.4) | 0.802 |
| γ-glutamyl transferase | 179.4 (19.0-755.0) | 232.6 (30.0-810.0) | 0.640 |
| AST | 123.0(32.0-297.0) | 117.9 (45.0-305.0) | 0.897 |
| ALT | 223.2 (18.0-462.0) | 198.8 (45.0-398.0) | 0.665 |
| Total bilirubin | 40.3(6.7-94.6) | 31.4 (8.9-97.2) | 0.517 |
| Direct bilirubin | 19.6 (2.7-68.9) | 16.6 (4.2-51.5) | 0.719 |
| Total bile acid | 9.4 (3.0-15.1) | 11.5 (5.1-20.1) | 0.337 |
| Potassium | 3.9 (2.8-5.7) | 3.6 (1.6-4.9) | 0.506 |
| Urea nitrogen | 13.8 (3.1-30.6) | 11.8 (2.1-29.7) | 0.622 |
| Creatinine | 188.5 (62.3-363.4) | 160.6 (51.6-328.3) | 0.567 |
| Uric acid | 251.5 (52.4-402.7) | 207.4 (62.4-320.8) | 0.473 |
| PT | 17.0 (10.1-23.2) | 17.0 (10.2-22.9) | 0.988 |
| APTT | 42.3 (15.8-63.0) | 40.7 (18.9-64.8) | 0.833 |
| TT | 20.1 (8.9-28.0) | 19.2 (9.1-25.2) | 0.758 |
| INR | 1.5 (0.6-2.2) | 1.4 (0.7-2.3) | 0.571 |

**Supplementary Table 3. The sequences of siRNA、shRNA.**

| **si-NC** | sense 5’-UUCUUCGAACGUGUCACGUTT-3  anti-sense 5’-ACGUGACACGUUCGGAGAATT-3 |
| --- | --- |
| **si-TLN1** | sense 5’-GCUUCGAGCCCUACACUUUTT-3 anti-sense 5’-AAAGUGUAGGGCUCGAAGCTT-3 |
| **si-SMURF1** | sense 5’-GUGCCAUGAAAUGUUGAAUTT-3 anti-sense 5’-AUUCAACAUUUCAUGGCACTT-3 |
| **sh-EMP1** | GCCATCAAGGCTACTGTTTCA |
| **AAV-shEMP1** | GCCATCAAGGCTACTGTTTCA |
| **CON054** | TTCTCCGAACGTGTCACGT |
| **CON305** | CGCTGAGTACTTCGAAATGTC |

**Supplementary Table 4. List of antibodies used in the experiment.**

| **Antibody** | **Company** | **Cat#** |
| --- | --- | --- |
| **EMP1** | Abcam | ab202975 |
| **EMP1** | Bioss | bs-0558R |
| **EMP1** | Thermo Fisher | PA5-144812 |
| **TLN1** | Proteintech | 14168-1-AP |
| **FAK** | Proteintech | 12636-1-AP |
| **p-FAK** | Abcam | ab81298 |
| **p-FAK** | CST | 3283S |
| **AKT** | Proteintech | 10176-2-AP |
| **p-AKT** | Proteintech | 66444-1-Ig |
| **α-SMA** | Proteintech | 14395-1-AP |
| **COL1A2** | Proteintech | 14695-1-AP |
| **TGF-β1** | Proteintech | 26155-1-AP |
| **IL-1β** | ABclonal | A1112 |
| **TNF-α** | Proteintech | A11534 |
| **SMURF1** | Proteintech | 55175-1-AP |
| **Flag** | Abcam | ab205606 |
| **Myc** | CST | 2276T |
| **HA** | CST | 3724T |
| **ECM1** | Proteintech | 11521-1-AP |
| **CD44** | Proteintech | 60224-1-Ig |
| **CD31** | Proteintech | 85898-4-RR |
| **β-Tubulin** | Proteintech | 10094-1-AP |
| **GAPDH** | Proteintech | 60004-1-Ig |

**CST: Cell Signaling Technology**

**Supplementary Table 5. The primer sequences for RT-qPCR.**

| **Gene** | **Forward primer (5′ - 3′)** | **Reverse primer (5′ - 3′)** |
| --- | --- | --- |
| **Emp1 (rat)** | ATGGTGGCGGACGGTATAGACTC | AAGCAGAAGCAAATCCAGGTCAGG |
| **Tln1 (rat)** | TGTGATGCGTGTGGATGAGAAGAC | ACTCGGTTGTATTGCTGCTGAAGG |
| **Smurf1(rat)** | TGTGCGGTTGTATGTGAACTGGAG | TGTTGGCGTCAATCAGGTGGATG |
| **α-sma (rat)** | AGAACACGGCATCATCACCAACTG | TCACGCCATCTCCAGAGTCCAG |
| **Col1a2 (rat)** | CGAGGCAGAGATGGTGTTGATGG | TGAAGCCAGGAAGTCCAGGAGTC |
| **Tgf-β1(rat)** | GGCTGAACCAAGGAGACGGAATAC | GTGTGTCCAGGCTCCAAATGTAGG |
| **Tnf (rat)** | CTGGCGTGTTCATCCGTTCTCTAC | ACTACTTCAGCGTCTCGTGTGTTTC |
| **IL-1β (rat)** | ACAGCAATGGTCGGGACATAGTTG | TCAGAGGCAGGGAGGGAAACAC |
| **Ecm1(rat)** | CCAGCAGGGACGGAGAGGTATC | CAGCGACAGCAGCGGGAATATC |
| **Cd44 (rat)** | GCCCGCACAGAAGACAACCAG | CCCTGCCATCCGTTCTGAAACC |
| **Gapdh (rat)** | GTCCATGCCATCACTGCCACTC | CGCCTGCTTCACCACCTTCTTG |
